# Supplementary material for: Disentangling bacterial invasiveness from lethality in an experimental host‐pathogen system
Source: Mol Syst Biol. 2019 Jun 11;15(6):e8707. doi: 10.15252/msb.20188707 (PMC6558951; doi:10.15252/msb.20188707)
Supplement: Supplementary file 1 — Appendix [file MSB-15-e8707-s001.pdf]

Disentangling bacterial invasiveness from lethality in an experimental host-pathogen system (Supplementary Material: Theoretical Model)

Tommaso Biancalani and Jeff Gore
Physics of Livings Systems, Department of Physics, Massachusetts Institute of Technology, Cambridge, Massachusetts, United States of America

I. MODEL DEFINITION AND ANALYSIS

Here, we provide more detail about the derivation of the model equations (*i.e.* equations (1) and (2) in the main text) and the explicit solutions that we used to carry out a non-linear fit of our data.

A. Equation for pathogen growth in worm

To model the pathogen dynamics inside the worm gut, we assume that the pathogen abundance per worm is equal at all times across the worm population. This assumption is justified by the fact that in our experiments each worm is treated in the same way, although we are neglecting stochastic effects that can be present during colonization or infection. We denote by $x(t)$ the pathogen abundance in a single worm at time t in carrying capacity units, and suppose that $x(t)$ is governed by the following equation:

$$\dot{x}(t) = (rx + c)\,(1 - x)\,. \tag{1}$$

This model describes the following dynamics: the pathogen colonizes the host from the external environment with a constant colonization rate c, and replicates inside the host at growth rate r until it reaches carrying capacity $x = 1$. If we switch off external colonization, $c = 0$, Eq. (1) reduces to the classical logistic growth equation. Another possible choice for our model would be the equation $\dot{x}(t) = rx\,(1-x) + c$. The two equations lead to similar results, although the analytical treatment is simpler for Eq. (1). In addition, Eq. (2) ensures that when the pathogen is at carrying capacity, no colonization or replication can occur, which would have not be true if we had instead chosen the model $\dot{x}(t) = rx\,(1-x) + c$. Equation (1) in this SM becomes equation (1) in the main text by setting $x = N/K$, and by rescaling the colonization rate by $c \mapsto c \cdot K$. The latter rescaling is used to set the units of measure of the colonization rate to $\mathrm{hr}^{-1} \cdot$cells which bears a more sound biological meaning.

Since each worm is sterile at the beginning of the experiment, we set our initial condition to be $x(0) = 0$. We can then solve Eq. (1) by direct integration with respect to time t to obtain $t(x)$. Inverting the function $t(x)$ and setting $x(0) = 0$ yields the solution of the model:

$$x(t) = \frac{e^{t/\sigma} - 1}{e^{t/\sigma} + \frac{r}{c}}, \quad \text{where} \quad \sigma = \frac{1}{r + c}. \tag{2}$$

We used the above formula to fit the data in Figure 3 and Figure 4-C in the main text. This formula shows that the timescale for the system is given by σ, the sum of the colonization and the growth rates. For $r = c$, Eq. (2) reduces to $x(t) = \tanh(t/\sigma)$, which is a popular model for sigmoidal growth in population dynamics. More generally, this model describes a monotonic increase in pathogen abundance to the carrying capacity value $x = 1$ and, indeed, when t is large the exponential in Eq. (2) dominates showing that $x(t) \sim 1$. For early times, we can approximate the exponentials in Eq. (2) using $e^{t/\tau} \sim 1 + t/\tau$, and arrive to

$$x(t) \sim \frac{c\,t}{1 + c\,t}, \quad \text{when } t \sim 0, \tag{3}$$

which shows that colonization is the sole effect in play at initial times. Finally, Eq. (2) shows that rescaling colonization and growth rates is tantamount to an overall change in time scale. Curves from Eq. (2) are plotted in Fig. 1

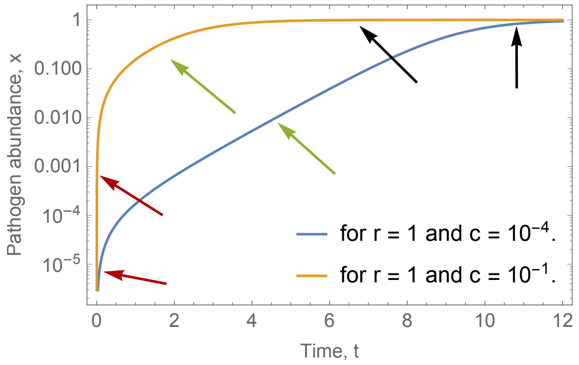

FIG. 1. **Pathogen growth curves exhibit three phases.** Pathogen abundance growth curves, as predicted by Eq. (2), displayed in semi-log scale for two instances of parameter values. Initially, colonization dominates (red arrows) followed by replication (green arrows), until the abundances saturate to carrying capacity (black arrows).

B. Equation for the fraction of worms surviving

We denote by $w(t)$ the fraction of worms surviving at time t. At the beginning of the experiment every worm is alive, so that we set $w(0) = 1$. Following the hypothesis described in the main text, we model the population death rate proportionally to the pathogen load in a linear

fashion:

$$\dot{w}(t) = -\delta\, x\, w, \tag{4}$$

where δ is the pathogen lethality.

Equation (4) states that each worm dies with rate $\delta x(t)$, whose value increases in time as the pathogen abundance grows in the population. The constant δ is required to dimensionalize the right-hand of the equation and sets the timescale on which the population decays. Plugging Eq. (2) into Eq. (5), and solving for w, we arrive at our prediction for the survival function, which we used to fit the data in Figure 3 in the main text:

$$w(t) = e^{\frac{\delta c}{r} t} \left(\frac{1 + \frac{r}{c}}{e^{(r+c)t} + \frac{r}{c}} \right)^{\frac{\delta}{r}}. \tag{5}$$

For large times, $x \sim 1$ and therefore $\dot{w} \sim \delta w$, thus indicating that the population copy number decays exponentially with exponent δ. Another way to see the effect is to directly approximate Eq. (5) for large times. The approximation reads:

$$w(t) \sim (r + c)^{\delta/r}\, e^{-\delta\, t}, \quad \text{for } t \gg 0. \tag{6}$$

The exponential decay of $w(t)$ can be graphically visualized plotting the quantity in semi-log scale, which yields a line with slope $\arctan(\delta)$. This also provides a convenient way to estimate δ from the data. An example of survival function from Eq. (5) is shown in Fig. 2.

II. INVASION TIME, COLONIZATION TIME AND REPLICATION TIME

We now derive the expressions for the invasion time τ, the colonization time τ_c and the replication time τ_r (corresponding to equations (3) and (4) in the main text).

We start by considering the invasion time. From Fig. 2, and also from Eq. (5), it is clear that for early times $w(0) \sim 1$. Therefore, it is convenient to define the invasion time τ as the time at which the line $w(0) = 1$ intercepts the line given by Eq. (6) displayed in semi-log scale. The invasion time is the reciprocal of the pathogen *invasiveness* and according to our model reads:

$$\tau = \frac{1}{r} \log\left(1 + \frac{r}{c}\right). \tag{7}$$

The expression found in the main text follows by rescaling the above equation by $c \mapsto c \cdot K$.

Let us now consider Eq. (2). At early times $x \ll 1$, so that the saturation term can be neglected and Eq. (2) becomes $\dot{x} \approx rx + c$. If also $x \ll c/r$, then the equation simplifies further to $\dot{x} \approx c$ showing that external colonization is the only relevant effect. Instead, if $1 \gg x \gg c/r$, the equation for the pathogen load becomes $\dot{x} \approx rx$ so that now colonization can be neglected. As the pathogen grows in the population, saturation becomes more relevant and the effective equation should be corrected to $\dot{x} \approx rx(1 - x)$.

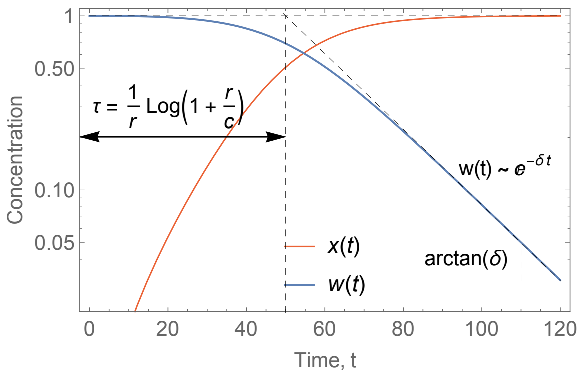

FIG. 2. **Our model disentangles pathogen invasiveness from lethality.** Pathogen invasiveness, τ^{-1}, and lethality, δ, as predicted by our model. Solid lines correspond to the pathogen growth curve Eq. (2) (red), and the survival function Eq. (5) (blue), obtained for parameters $\delta = 1/20$, $c = 10^{-3}$ and $r = 9/100$.

Our reasoning indicates that there is a transition between early times, where colonization dominates, and late times, where colonization can be neglected. This transition is determined by the pathogen abundance reaching the critical value $x^* = c/r$. Using Eq. (2), we can solve for the time τ_c taken by the pathogen abundance to reach such critical value, *i.e.* $x(\tau_c) = x^*$:

$$\tau_c = \frac{1}{r + c} \log\left(\frac{2r}{r - c}\right). \tag{8}$$

To expression for the replication time, $\tau_r = \tau - \tau_c$ is obtained by subtracting Eq. (9) from Eq. (7):

$$\tau_r = \frac{1}{r + c} \log\left(\frac{r^2 - c^2}{2rc}\right). \tag{9}$$

To conclude, we note that if $c \ll r$, as occurs in our experiments, the colonization rate c can be neglected in the expression of the colonization time τ_c. Therefore the expression for the colonization time reduced to the that of the pathogen doubling time, which is given by equation (4) in the main text.

Statistical analysis for *Disentangling bacterial invasiveness from lethality in an experimental host-pathogen system*

Tommaso Biancalani and Jeff Gore

Clear environment and set working directories (set your own path).

```
rm(list=ls())
wd1 <- '/Users/tbiancal/git/disentangling-pathogens-SM/exp1/'
wd2 <- '/Users/tbiancal/git/disentangling-pathogens-SM/exp2/'
```

Linear fit for lethalities δ in survival curves in Fig. 1-A and Fig. S1

Load file lists of each experimental condition.

```
paA = list.files(path=wd2, pattern="paA[[:alpha:]]")
paB = list.files(path=wd2, pattern="paB[[:alpha:]]")
paC = list.files(path=wd2, pattern="paC[[:alpha:]]")
smA = list.files(path=wd2, pattern="smA[[:alpha:]]")
smB = list.files(path=wd2, pattern="smB[[:alpha:]]")
smC = list.files(path=wd2, pattern="smC[[:alpha:]]")
seA = list.files(path=wd2, pattern="seA[[:alpha:]]")
seB = list.files(path=wd2, pattern="seB[[:alpha:]]")
seC = list.files(path=wd2, pattern="seC[[:alpha:]]")
```

Load data from CSV files.

```
read_csv_list <- function(wd, csv_files) {
  # Read list of CSV files and return list of corresponding dataframes
  dfs <- list()
  for (csv_file in csv_files) {
    csv_file = paste(wd, csv_file, sep = "")
    df <- read.csv(csv_file)
    dfs <- c(dfs, list(df))
  }
  return(dfs)
}

PaA_dfs = read_csv_list(wd2, paA)
PaB_dfs = read_csv_list(wd2, paB)
PaC_dfs = read_csv_list(wd2, paC)

SmA_dfs = read_csv_list(wd2, smA)
SmB_dfs = read_csv_list(wd2, smB)
SmC_dfs = read_csv_list(wd2, smC)

SeA_dfs = read_csv_list(wd2, seA)
SeB_dfs = read_csv_list(wd2, seB)
SeC_dfs = read_csv_list(wd2, seC)
```

Normalize survival curves to get fraction of worms surviving on y-axis.

```r
normalize_survival_curves <- function (dfs) {
  # Take list of survival curves and return normalized list of survival curves

  norm_dfs <- list()
  for (df in dfs) {
    n_worms = df[[1, 2]]
    df[[2]] = df[[2]] / n_worms
    norm_dfs <- c(norm_dfs, list(df))
  }
  return(norm_dfs)
}

PaA_dfs = normalize_survival_curves(PaA_dfs)
PaB_dfs = normalize_survival_curves(PaB_dfs)
PaC_dfs = normalize_survival_curves(PaC_dfs)

SmA_dfs = normalize_survival_curves(SmA_dfs)
SmB_dfs = normalize_survival_curves(SmB_dfs)
SmC_dfs = normalize_survival_curves(SmC_dfs)

SeA_dfs = normalize_survival_curves(SeA_dfs)
SeB_dfs = normalize_survival_curves(SeB_dfs)
SeC_dfs = normalize_survival_curves(SeC_dfs)
```

Display mean survival curve to detect invasion time (vertical bar), which is used to determine the fitting region.

```r
display_mean_surv_curve <- function (dfs, title_text, num_pts) {
  # Display mean surv. curve averaged iover list of dataframes `dfs`
  # Set figure title to `title_text`
  # Draw vertical line on plot to separate last `num_pts`
  # Return `num_pts`

  ## Get mean survival curve
  w_rows <- list()
  for (df in dfs) {
    w_rows <- as.double(c(w_rows, df[[2]]))
  }
  row_matrix <- matrix(w_rows, nrow = length(dfs), byrow = TRUE)
  mean_sc <- colMeans(row_matrix)

  ## Plot
  times <- dfs[[1]][[1]]
  xlab <- 'Time (hr)'
  ylab <- 'Fraction of worms surviving'
  plot(times, mean_sc, log = 'y', type = 'b', main = title_text, xlab = xlab, ylab = ylab)

  ## Draw vertical line
  threshold_time <- rev(times)[[num_pts]]
  abline(v=threshold_time)

  return(num_pts)
}
```

For *P. aeruginosa*:

```
PaA_npts <- display_mean_surv_curve(PaA_dfs, 'Pa 48h', 3)
```

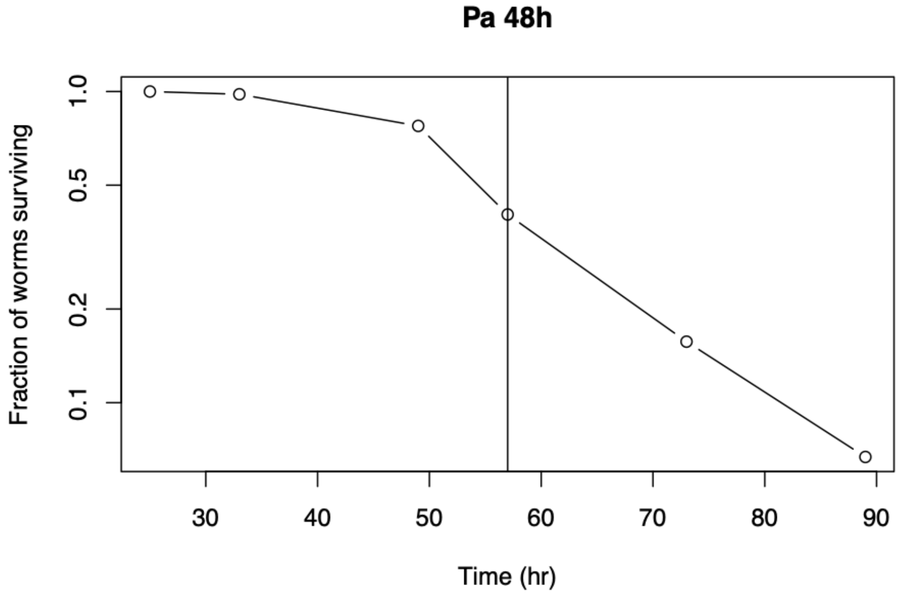


```
PaB_npts <- display_mean_surv_curve(PaB_dfs, 'Pa 24h', 4)
```

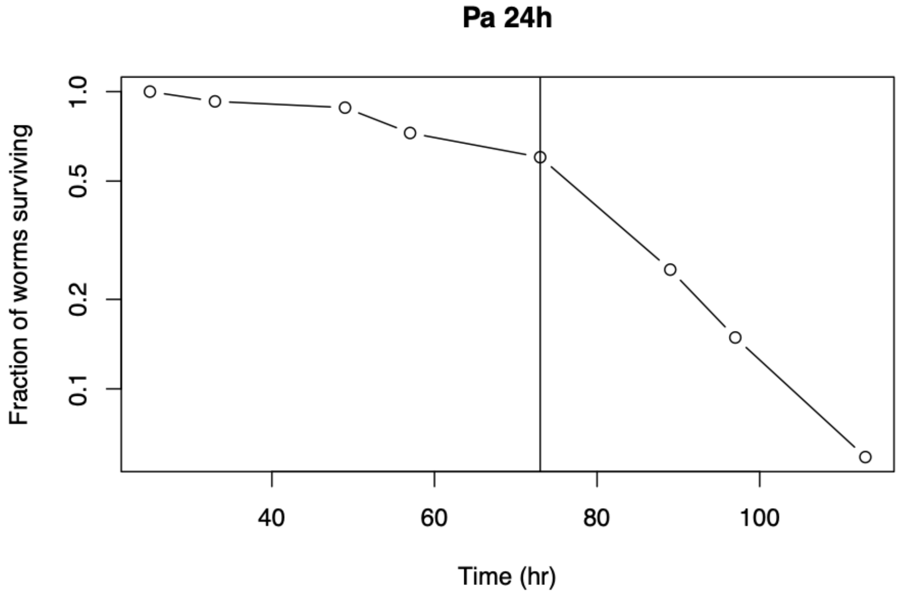

```r
PaC_npts <- display_mean_surv_curve(PaC_dfs, 'Pa 4h', 4)
```

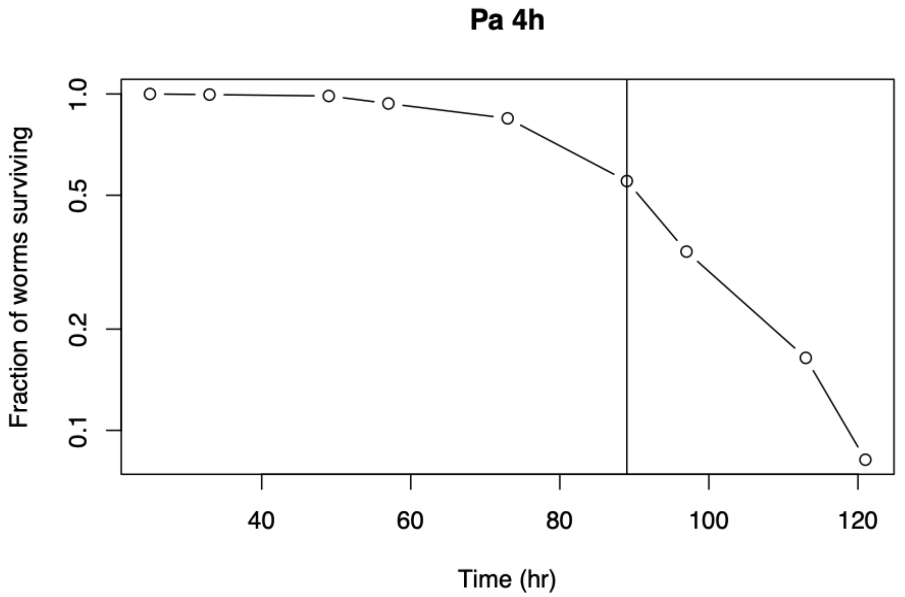

For *S. marcescens*:

```r
SmA_npts <- display_mean_surv_curve(SmA_dfs, 'Sm 48h', 3)
```

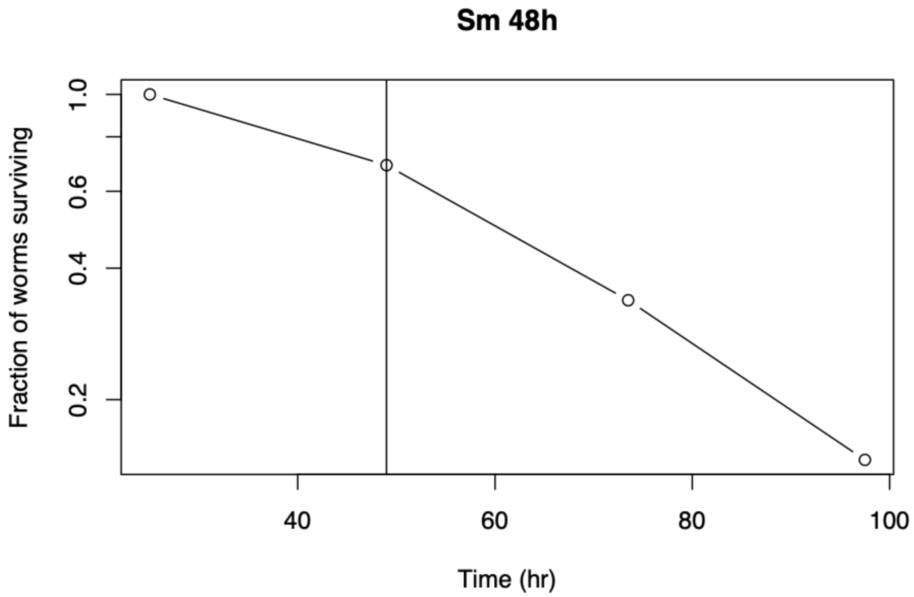

```
SmB_npts <- display_mean_surv_curve(SmB_dfs, 'Sm 24h', 3)
```

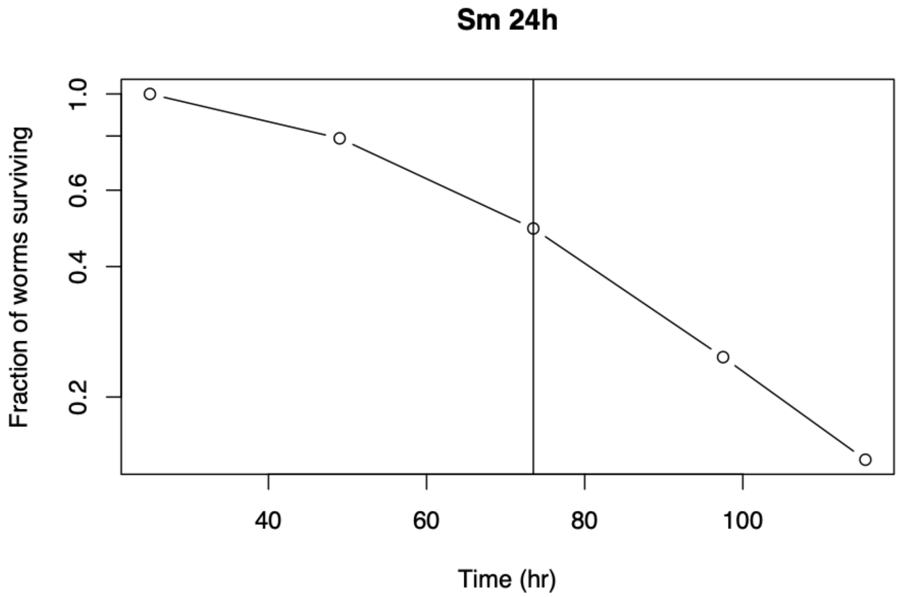


```
SmC_npts <- display_mean_surv_curve(SmC_dfs, 'Sm 4h', 3)
```

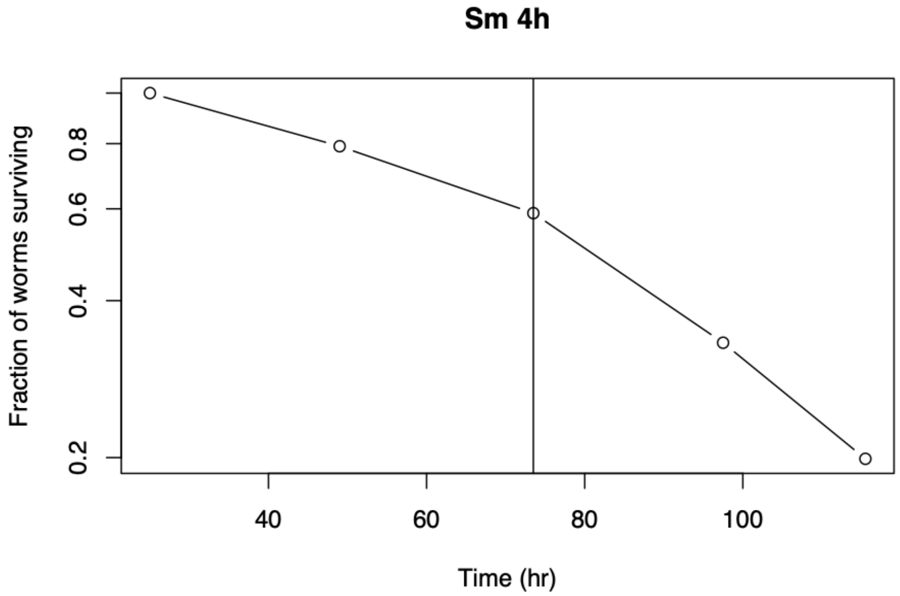

For *S. enterica*:

```r
SeA_npts <- display_mean_surv_curve(SeA_dfs, 'Se 48h', 3)
```

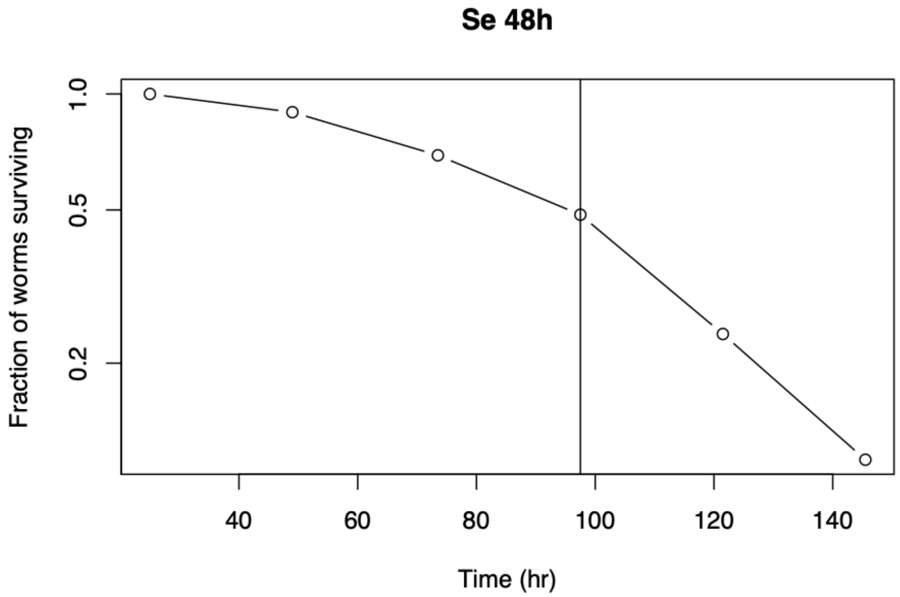


```r
SeB_npts <- display_mean_surv_curve(SeB_dfs, 'Se 24h', 3)
```

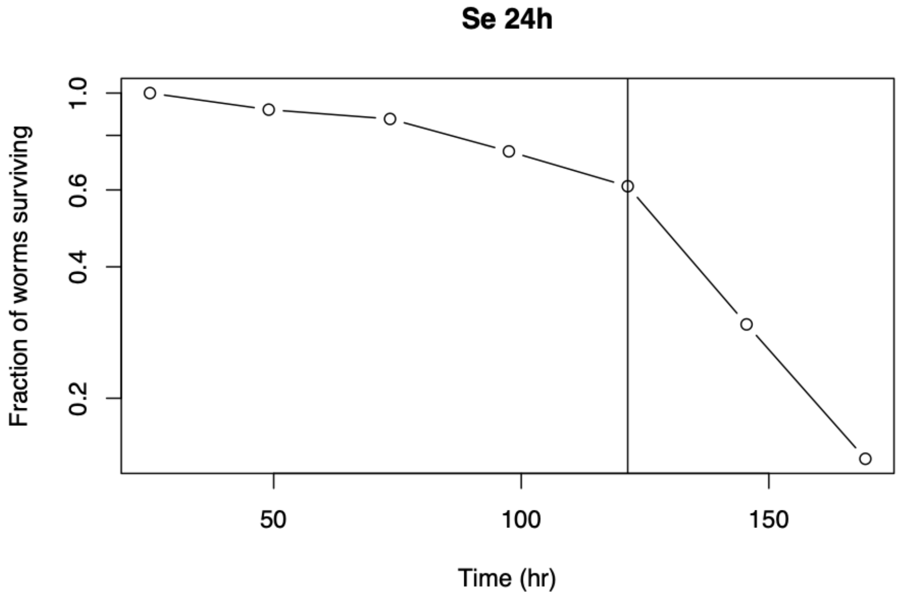

```r
SeC_npts <- display_mean_surv_curve(SeC_dfs, 'Se 4h', 3)
```

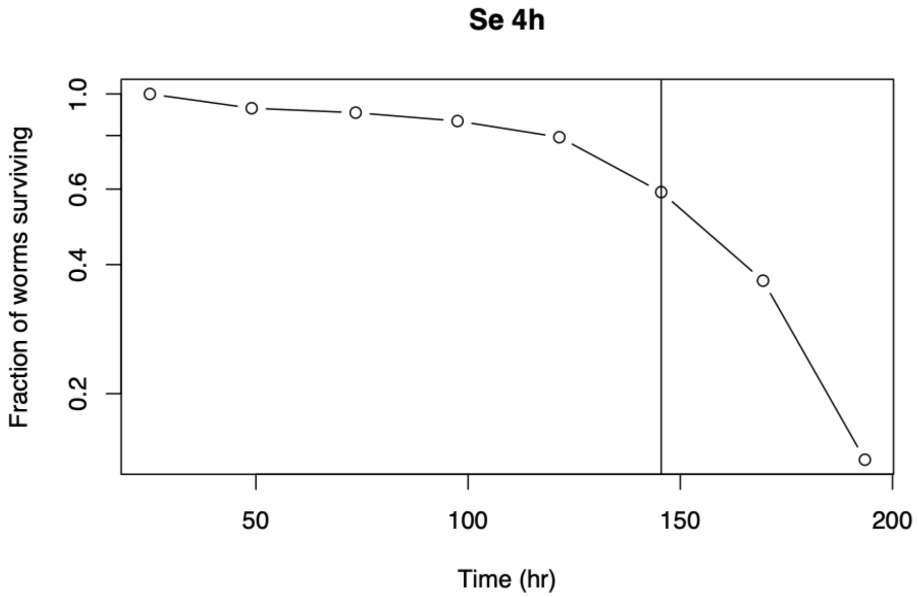

Fit lethality to each survival curve using a linear model. The fitting region is determined by the invasion times from the mean survival curves.

```r
find_models <- function (dfs, num_pts) {
  # Take last `num_pts` from each df from `dfs,
  # Compute log of y and perfom linear fit.
  # return fitted models
  models <- list()
  for (df in dfs) {

    ## Convert to semi-log
    last_pts = tail(df, num_pts)
    t <- last_pts[[1]]
    w <- log(last_pts[[2]])

    ## Purge non-valid values
    mask <- is.finite(w)
    w <- w[mask]
    t <- t[mask]

    ## Linear fit
    model <- lm(w ~ t)
    models <- c(models, list(model))
  }
  return(models)
}

PaA_models = find_models(PaA_dfs, PaA_npts)
```

```r
PaB_models = find_models(PaB_dfs, PaB_npts)
PaC_models = find_models(PaC_dfs, PaC_npts)

SmA_models = find_models(SmA_dfs, SmA_npts)
SmB_models = find_models(SmB_dfs, SmB_npts)
SmC_models = find_models(SmC_dfs, SmC_npts)

SeA_models = find_models(SeA_dfs, SeA_npts)
SeB_models = find_models(SeB_dfs, SeB_npts)
SeC_models = find_models(SeC_dfs, SeC_npts)
```

Display linear fits.

```r
display_surv_fit <- function (dfs, models, title_text) {
  # Display overlayed surv. curves. from list of dataframes `dfs`
  # Set figure title to `title_text`
  # Overlay linear fit from `models`.

  ## Main plot
  df <- dfs[[1]]
  title <- title_text
  xlab <- 'Time (hr)'
  ylab <- 'Fraction of worms surviving'
  t <- df[[1]]
  w <- log(df[[2]])
  options <- list(
    t, w, col=1, type='p', main=title, xlab=xlab, ylab=ylab
    )
  do.call(plot, options)

  ## Overlay secondary plots
  for (i in 2:length(dfs)) {
    df <- dfs[[i]]
      t <- df[[1]]
    w <- log(df[[2]])
    points(t, w, col=i, type='p')
  }

  ## Overlay linear fit
  for (i in 1:length(models)) {
    model <- models[[i]]
    t <- df[[1]]
    w <- log(df[[2]])
    col <- i
    abline(model, col=i, lwd=.6, lt=2)
  }
}

display_surv_fit(PaA_dfs, PaA_models, "Pa 48h")
```

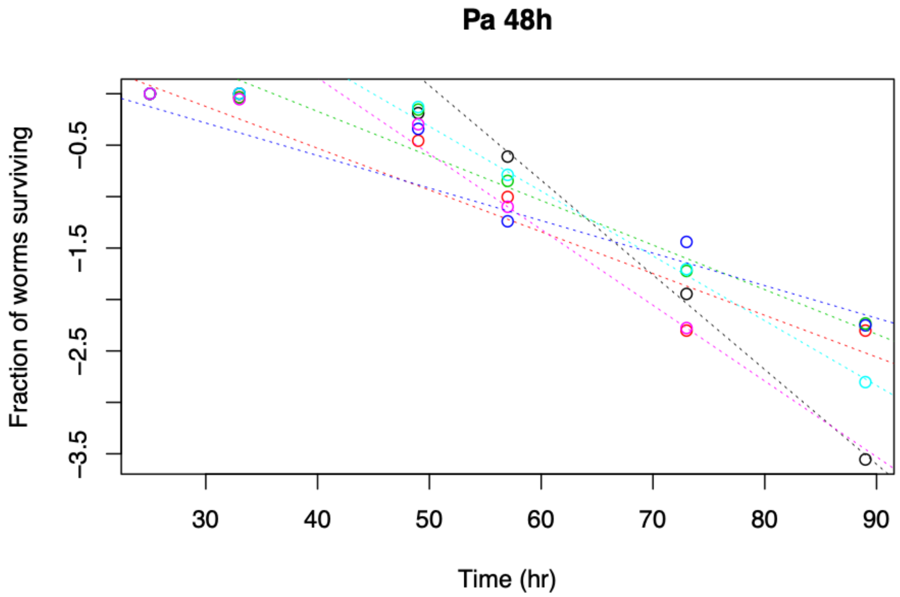


```r
display_surv_fit(PaB_dfs, PaB_models, "Pa 24h")
```

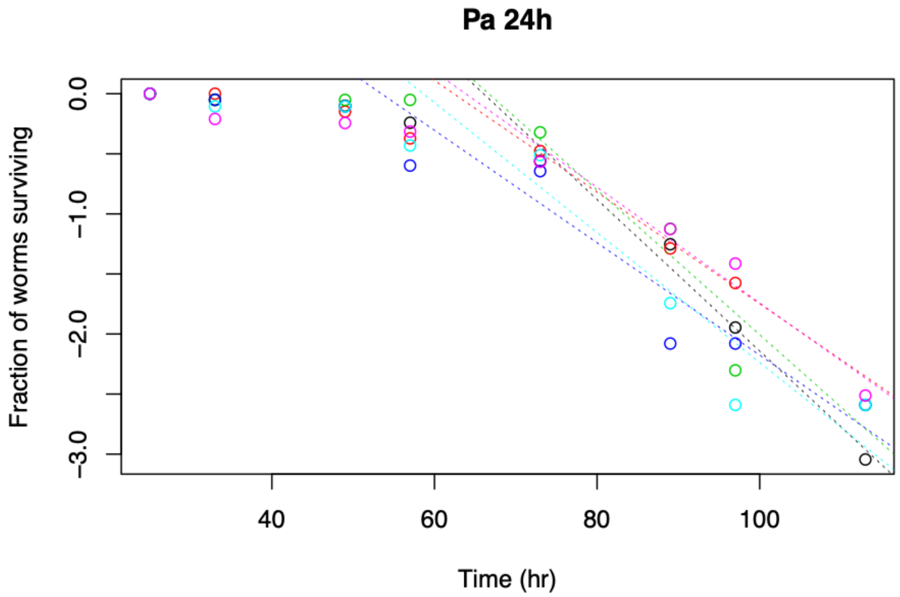


```r
display_surv_fit(PaC_dfs, PaC_models, "Pa 4h")
```

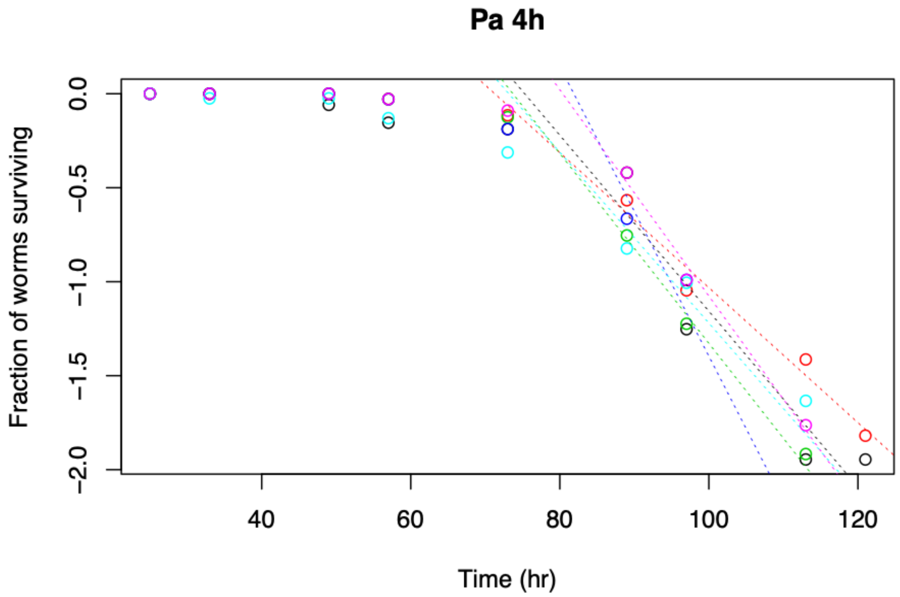


```
display_surv_fit(SmA_dfs, SmA_models, "Sm 48h")
```

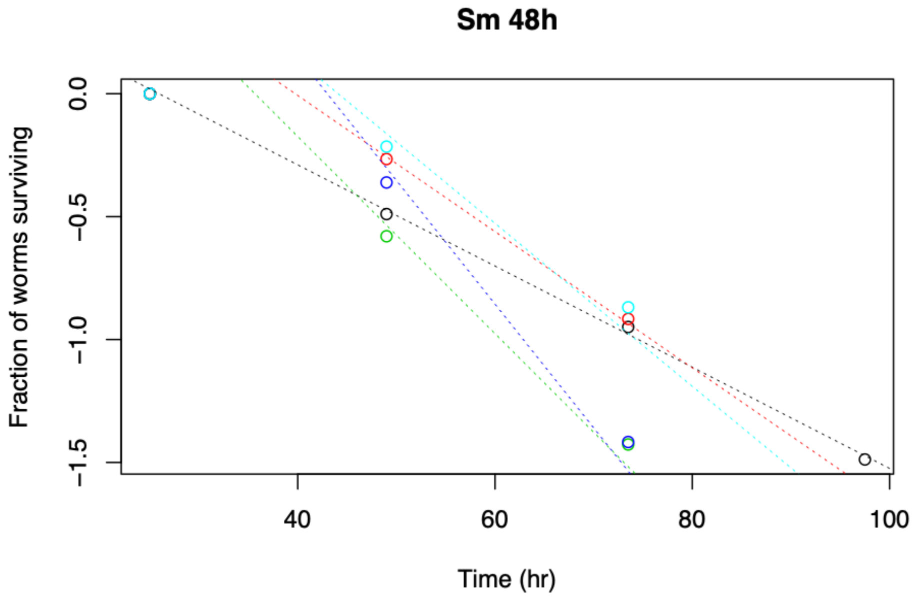


```
display_surv_fit(SmB_dfs, SmB_models, "Sm 24h")
```

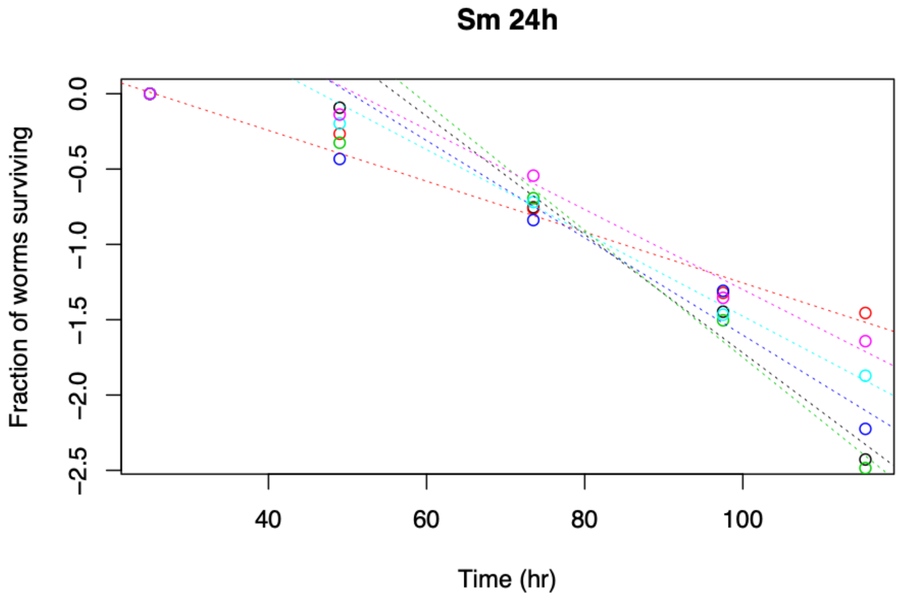


```r
display_surv_fit(SmC_dfs, SmC_models, "Sm 4h")
```

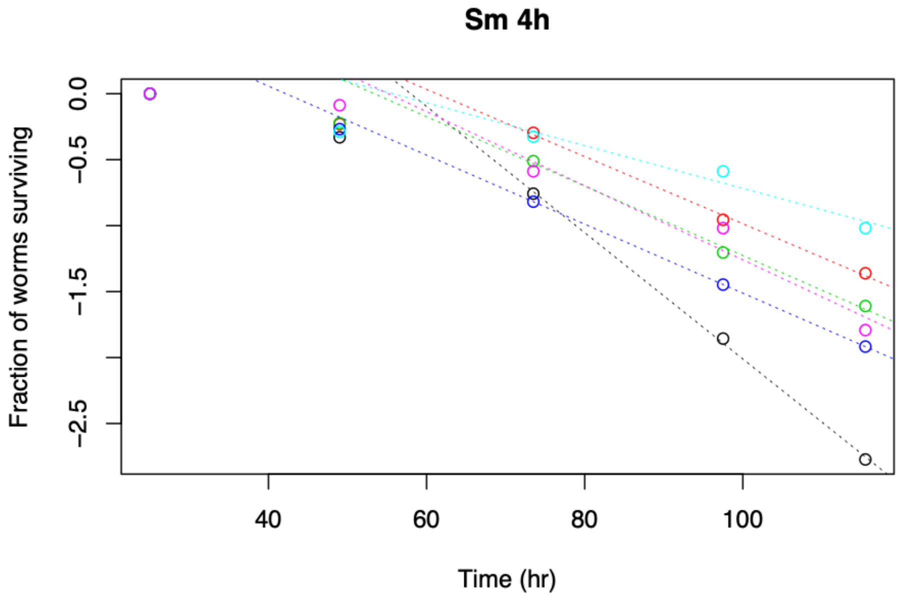


```r
display_surv_fit(SeA_dfs, SeA_models, "Se 48h")
```

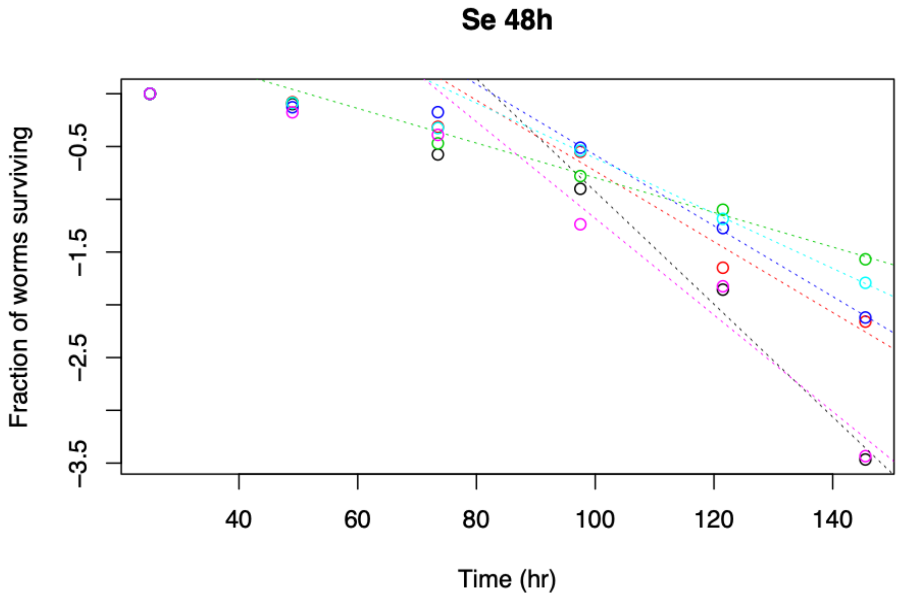


```r
display_surv_fit(SeB_dfs, SeB_models, "Se 24h")
```

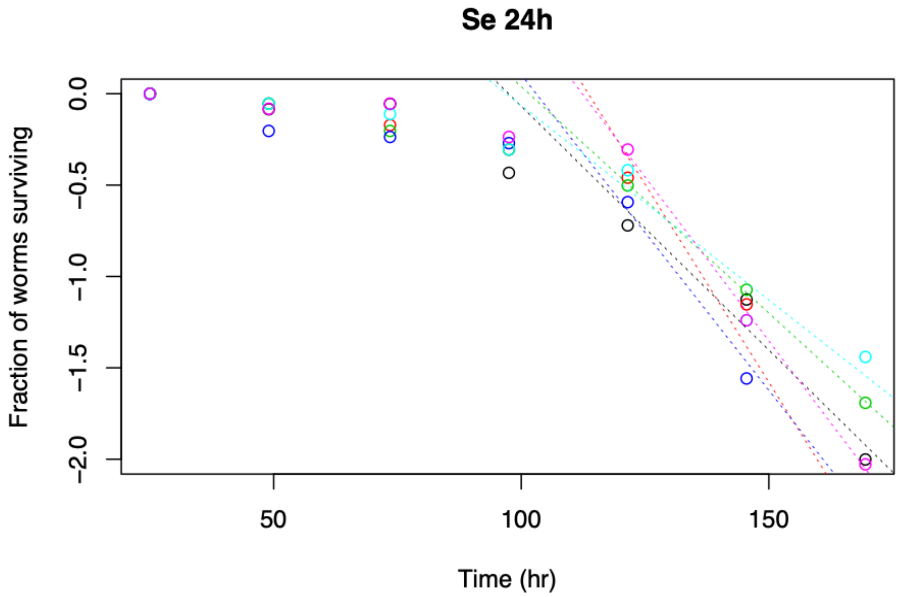


```r
display_surv_fit(SeC_dfs, SeC_models, "Se 4h")
```

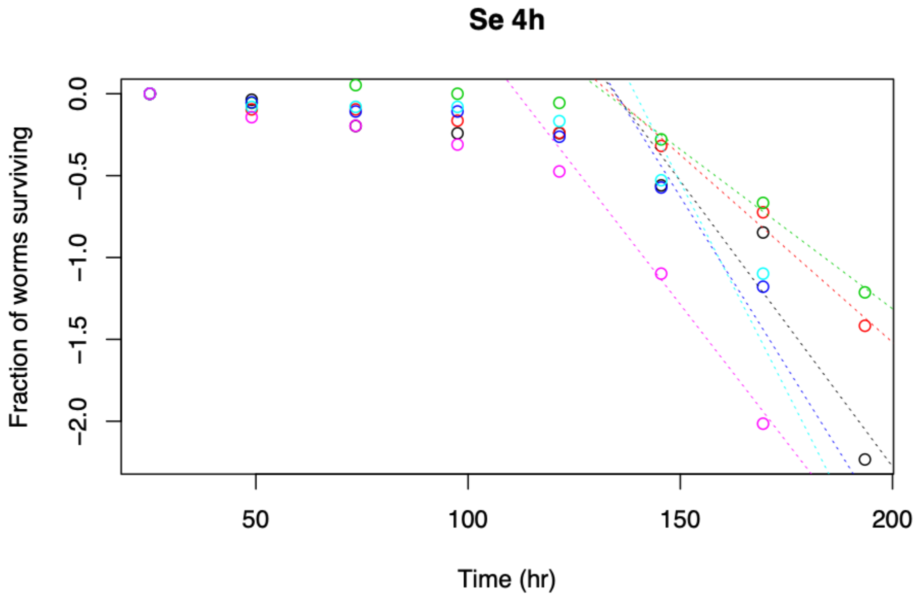

Compute mean lethalities and their standard errors.

```
get_delta_w_err <- function (models) {
  deltas <- list()
  for (model in models) {
    delta <- model$coefficients[[2]]
    deltas <- c(deltas, delta)
  }
  deltas <- unlist(deltas)  # cast to vector
  delta <- -mean(deltas)
  delta <- round(delta, digits = 3)
  err <- sqrt(var(deltas)/length(deltas))
  err <- round(err, digits = 3)
  return (c(delta, err))
}

PaA_delta  <- get_delta_w_err(PaA_models)
PaB_delta  <- get_delta_w_err(PaB_models)
PaC_delta  <- get_delta_w_err(PaC_models)

SmA_delta  <- get_delta_w_err(SmA_models)
SmB_delta  <- get_delta_w_err(SmB_models)
SmC_delta  <- get_delta_w_err(SmC_models)

SeA_delta  <- get_delta_w_err(SeA_models)
SeB_delta  <- get_delta_w_err(SeB_models)
SeC_delta  <- get_delta_w_err(SeC_models)

Pa_delta_df= data.frame(c("average lethality", "standard error"), PaA_delta, PaB_delta, PaC_delta, row.[illegible]
colnames(Pa_delta_df) <- c("Pa 48 hr", "Pa 24 hr", "Pa 4 hr")
```

```
Pa_delta_df
```

```
##                   Pa 48 hr Pa 24 hr Pa 4 hr
## average lethality    0.057    0.053   0.052
## standard error       0.009    0.003   0.006
```

```
Sm_delta_df= data.frame(c("average lethality", "standard error"), SmA_delta, SmB_delta, SmC_delta, row.
colnames(Sm_delta_df) <- c("Sm 48 hr", "Sm 24 hr", "Sm 4 hr")
Sm_delta_df
```

```
##                   Sm 48 hr Sm 24 hr Sm 4 hr
## average lethality    0.034    0.031   0.028
## standard error       0.005    0.004   0.004
```

```
Se_delta_df= data.frame(c("average lethality", "standard error"), SeA_delta, SeB_delta, SeC_delta, row.
colnames(Se_delta_df) <- c("Se 48 hr", "Se 24 hr", "Se 4 hr")
Se_delta_df
```

```
##                   Se 48 hr Se 24 hr Se 4 hr
## average lethality    0.035    0.031   0.034
## standard error       0.005    0.003   0.005
```

Linear fit for lethalities δ in survival curves in Fig. 1-B and Fig. S2

Find file list corresponding to technical replica

```
pa_fls = list.files(path=wd1, pattern="paW[[:alnum:]]")
sm_fls = list.files(path=wd1, pattern="smW[[:alnum:]]")
se_fls = list.files(path=wd1, pattern="seW[[:alnum:]]")
ph_fls = list.files(path=wd1, pattern="phW[[:alnum:]]")
```

Compute normalized survival curves from CSV files.

```
pa_dfs = read_csv_list(wd1, pa_fls)
pa_dfs = normalize_survival_curves(pa_dfs)

sm_dfs = read_csv_list(wd1, sm_fls)
sm_dfs = normalize_survival_curves(sm_dfs)

se_dfs = read_csv_list(wd1, se_fls)
se_dfs = normalize_survival_curves(se_dfs)

ph_dfs = read_csv_list(wd1, ph_fls)
ph_dfs = normalize_survival_curves(ph_dfs)
```

Display survival groups and determine threshold of exponential phase to be used for fitting.

```
pa_npts <-  display_mean_surv_curve(pa_dfs, "Pa", 4)
```

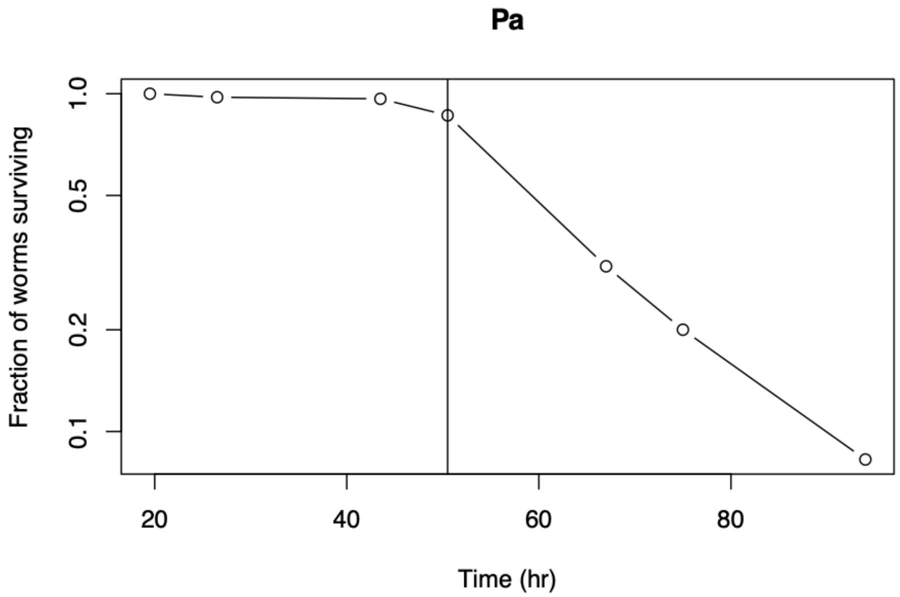


```
sm_npts <- display_mean_surv_curve(sm_dfs, "Sm", 5)
```

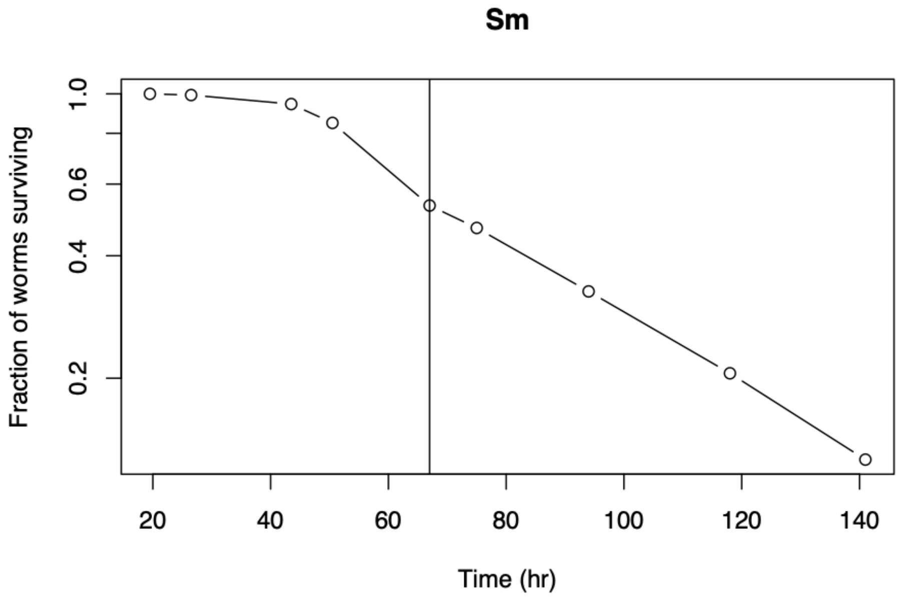


```
se_npts <- display_mean_surv_curve(se_dfs, "Se", 4)
```

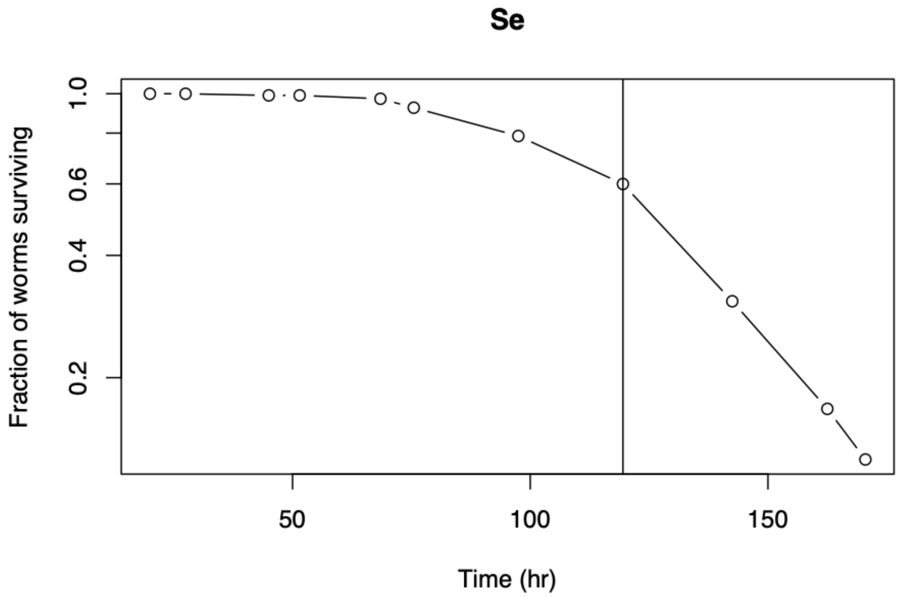


```
ph_npts <- display_mean_surv_curve(ph_dfs, "Ph", 4)
```

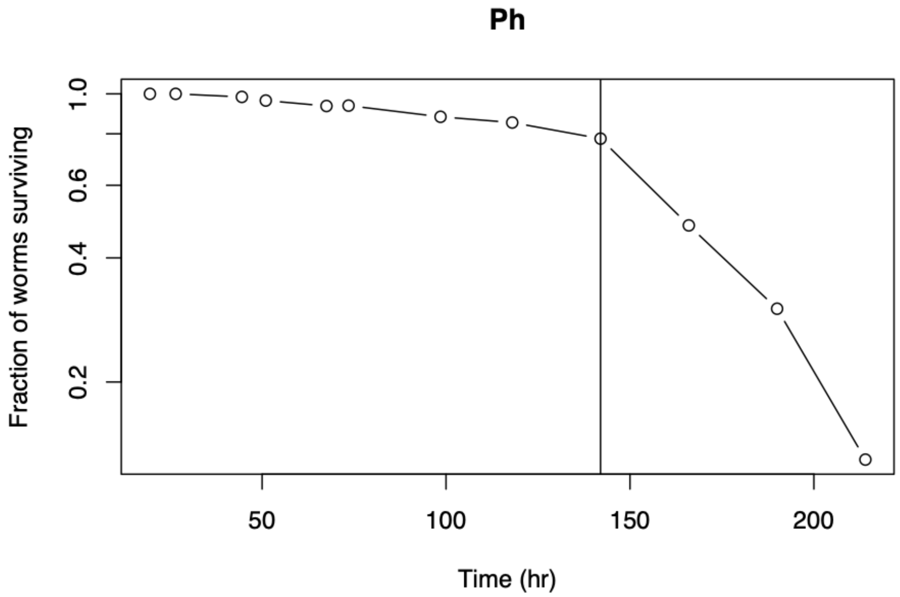

Fit lethality with linear model using thresholds previously found.

```
pa_models = find_models(pa_dfs, pa_npts)
sm_models = find_models(sm_dfs, sm_npts)
```

```r
se_models = find_models(se_dfs, se_npts)
ph_models = find_models(ph_dfs, ph_npts)
```

Check that fits are performed correctly.

```r
display_surv_fit(pa_dfs, pa_models, "Pa")
```

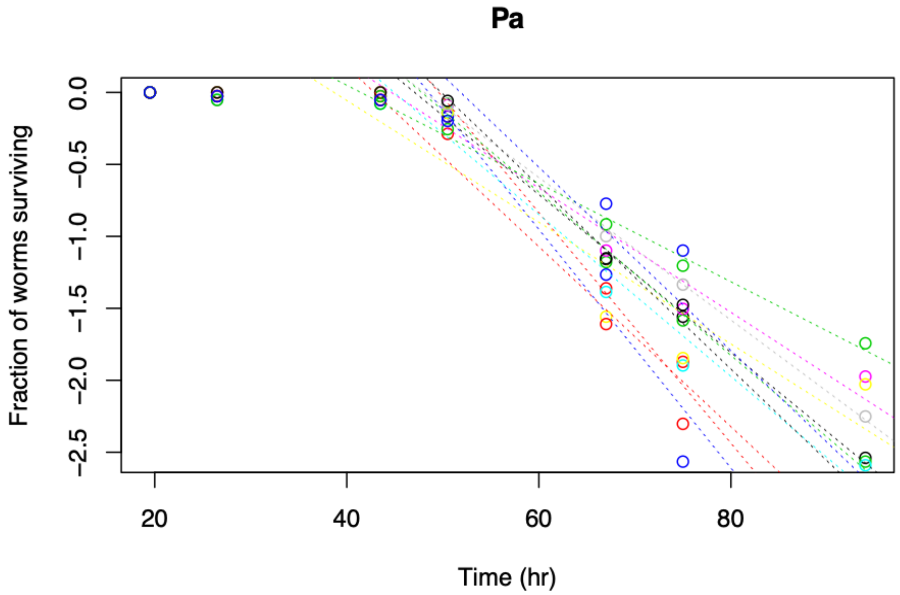


```r
display_surv_fit(sm_dfs, sm_models, "Sm")
```

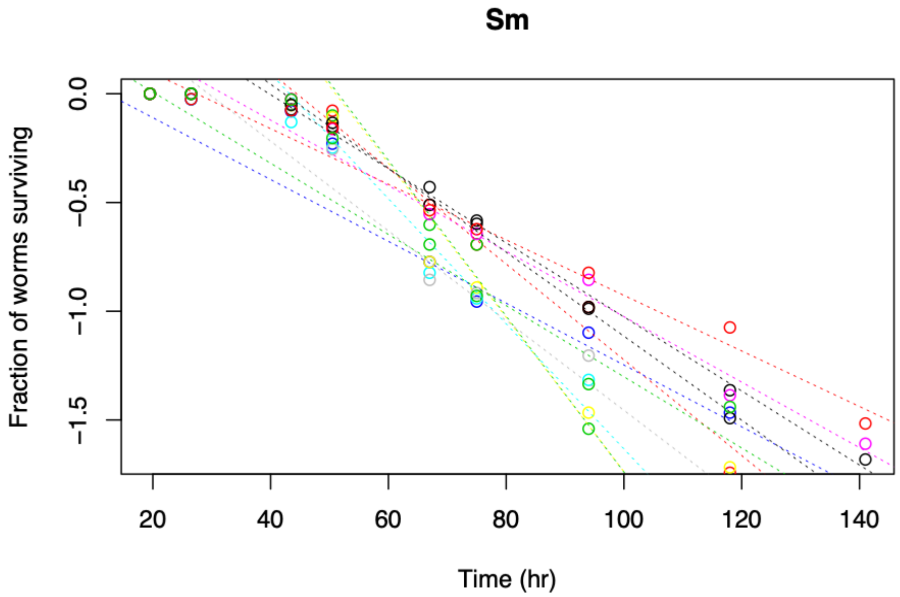


```
display_surv_fit(se_dfs, se_models, "Se")
```

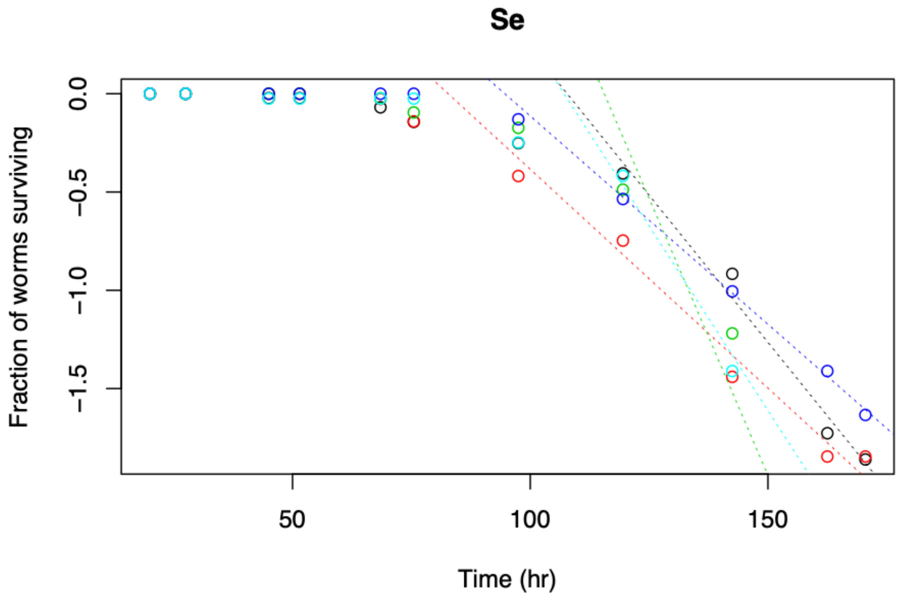


```
display_surv_fit(ph_dfs, ph_models, "Ph")
```

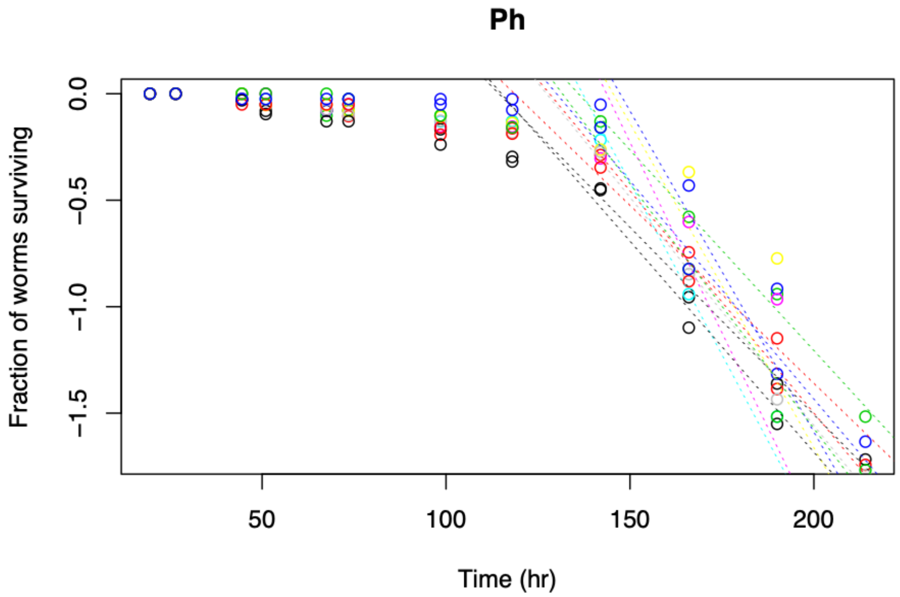

Compute average lethalities with s.e.m

```
pa_delta  <- get_delta_w_err(pa_models)
sm_delta  <- get_delta_w_err(sm_models)
se_delta  <- get_delta_w_err(se_models)
ph_delta  <- get_delta_w_err(ph_models)

res <- data.frame(c("average lethality", "standard error"), pa_delta, sm_delta, se_delta, ph_delta, row
colnames(res) <- c("Pa", "Sm", "Se", "Ph")
res
```

```
##                      Pa    Sm    Se    Ph
## average lethality 0.058 0.022 0.033 0.024
## standard error    0.004 0.002 0.006 0.002
```

Non-linear fit of *Pa* growth curves in Fig. 4-C

Fit model to mean *Pa* growth curves in Fig. 4-C

Load growth curves from CSV files

```
options <- list(
            header = FALSE,
            col.names =  c("times hr","Cells replica 1","Cells replica 2","Cells replica 3","Cells repl:
            )

args <- c(paste(wd2, 'paA_growth.csv', sep = ""), options)
paA_growth <- do.call(read.csv, args)

args <- c(paste(wd2, 'paB_growth.csv', sep = ""), options)
```

```r
paB_growth <- do.call(read.csv, args)

args <- c(paste(wd2, 'paC_growth.csv', sep = ""), options)
paC_growth <- do.call(read.csv, args)
```

Display data and mean curve of pathogen loads. Draw horizontal line to find carrying capacity.

```r
display_growth_data <- function (dfs, title_text, K) {
  # Print growth data from `dfs` along with mean curve.
  # Title figure `title_text`.
  # Draw horizontal line with intercept `K`
  # Return normalized mean curve as 2d list (times, vals).

  ## Get data as matrices
  cells <- as.matrix(dfs[, 2:5])
  ts <- matrix(rep(dfs[[1]], 4), nrow = 4, byrow = TRUE)
  ts <- t(ts)

  ## Compute mean curve
  mean_curve_cells <- rowMeans(cells, na.rm = TRUE)
  mean_curve_ts <- ts[,1]

  ## Flatten data for plotting
  ts <- as.vector(ts)
  cells <- as.vector(cells)

  ## Plot raw data
  plot(ts, cells,
       xlim = c(0, 120),
       ylim = c(10**2, 10**6),
       log = "y",
       main = title_text,
       xlab = 'Time (hr)',
       ylab = 'Pathogen load (cells)'
       )

  ## Add mean curve to plot
  points(mean_curve_ts, mean_curve_cells, type = 'l')

  ## Add carrying capacity line
  abline(h = K, lt=2, col='red')

  return(list(mean_curve_ts, mean_curve_cells / K))
}

KPa <- 2.8 * 10**5
paA_growth_norm <- display_growth_data(paA_growth, "Pa 48 hr", KPa)
```

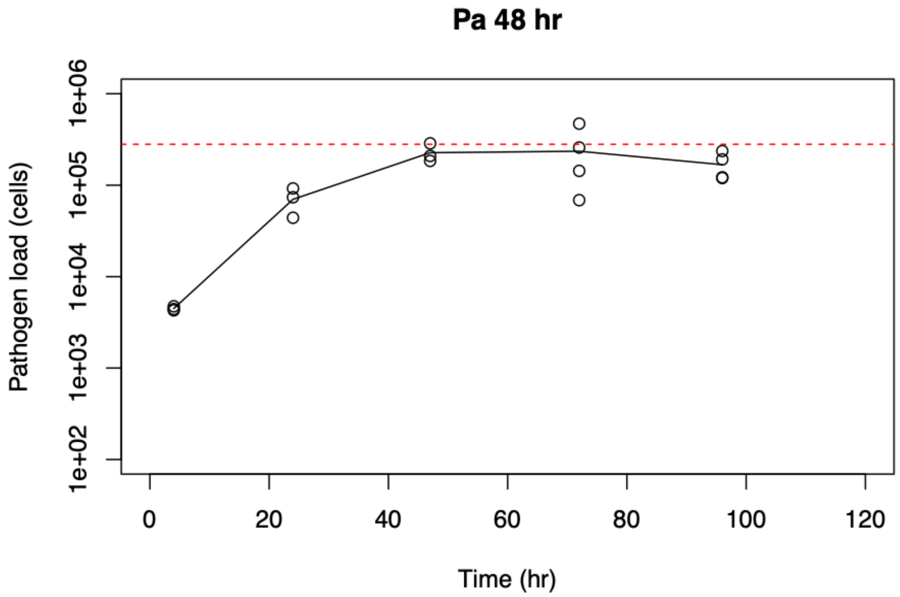


```
paB_growth_norm <- display_growth_data(paB_growth, "Pa 24 hr", KPa)
```

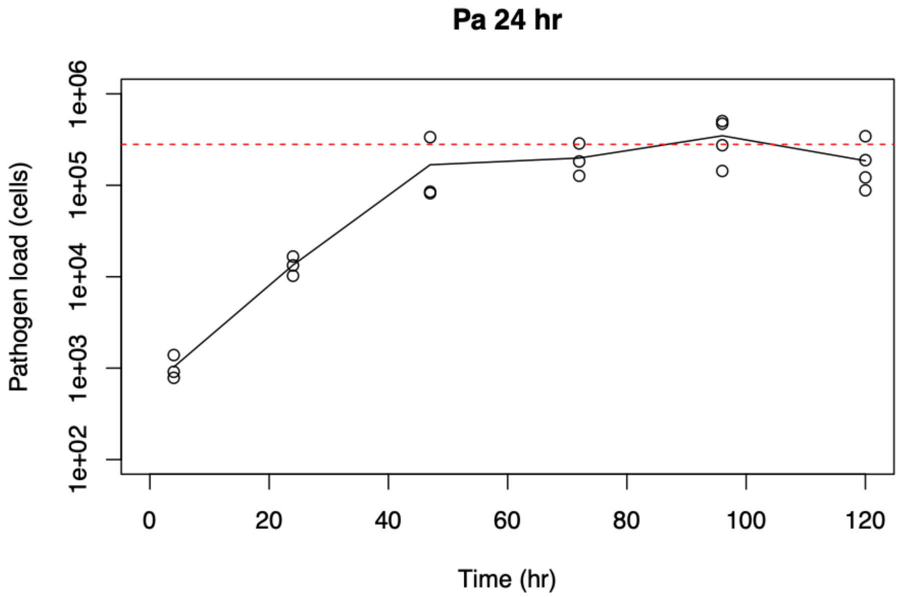


```
paC_growth_norm <- display_growth_data(paC_growth, "Pa 4 hr", KPa)
```

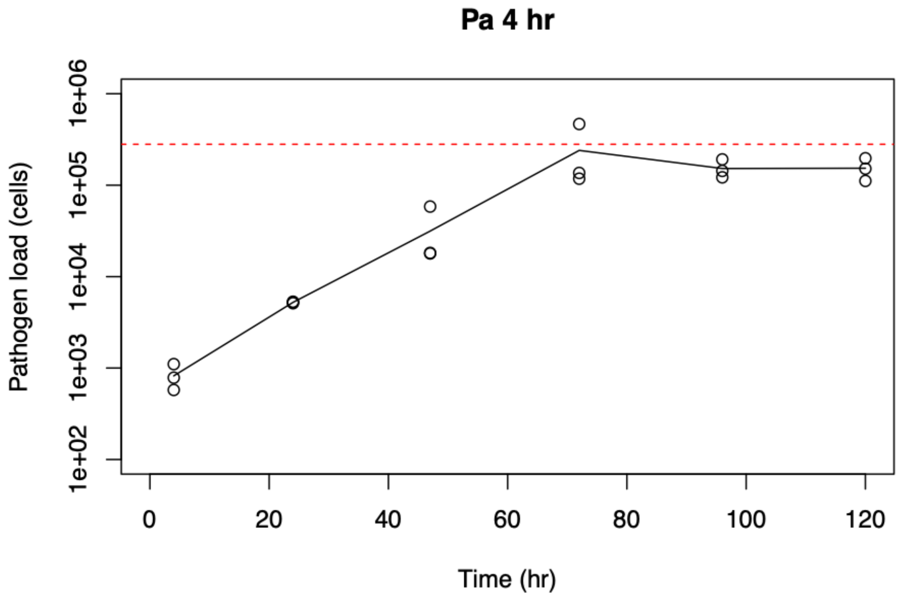

Compute model solution for pathogen load

```
evaluate_growth_model <- function (t, logr, logc) {
  # Return pathogen load at time t given growth rate rand colonization rate c.
  # `t` can be a list of times.
  # log transform r and c so that optimization problem is unconstrained.
  library("Brobdingnag")  # Handles very large number
  l <- as.brob(exp(logr) + exp(logc))
  # print(l)
  num <- exp(l*t) - 1
  # print(num)
  den <- exp(l*t) + as.brob(exp(logr)/exp(logc))
  # print(den)
  ans <- as.double(num / den)
  return (ans)
}
```

Plot growth data and model solution for some parameter values. These parameters will be used as starting parameters values for our fitting procedure.

```
library("pracma")
ts <- linspace(1, 120, n=100)
paA_model_dyn <- evaluate_growth_model(ts, log(0.08), log(0.0038210))
```

```
##
## Attaching package: 'Brobdingnag'

## The following objects are masked from 'package:base':
##
##     max, min, prod, range, sum
```

```
paB_model_dyn <- evaluate_growth_model(ts, log(0.08), log(7e-04))
paC_model_dyn <- evaluate_growth_model(ts, log(0.08), log(0.00019))

plot(ts, paA_model_dyn, type='l', col=2,
     main='Pa growth (model and data)',
     log='y', ylim=c(10**-4, 1),
     ylab="Pathogen load (fraction)",
     xlab="Time (hr)")

points(ts, paB_model_dyn, type='l', col=3)
points(ts, paC_model_dyn, type='l', col=4)

points(paA_growth_norm[[1]], paA_growth_norm[[2]], col=2, type = 'p')
points(paB_growth_norm[[1]], paB_growth_norm[[2]], col=3, type = 'p')
points(paC_growth_norm[[1]], paC_growth_norm[[2]], col=4, type = 'p')
```

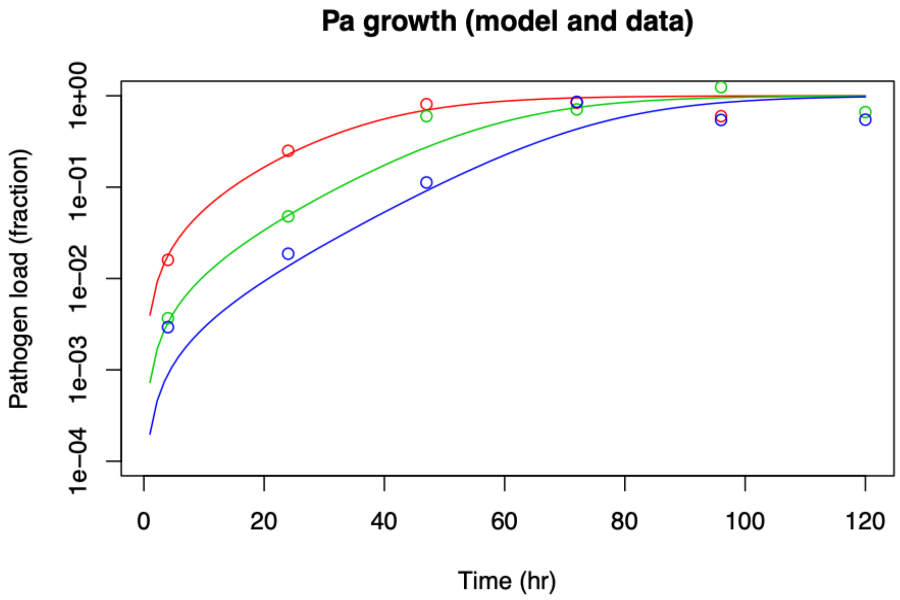

Compute sum-of-squares error between model solution and a single data set.

```
get_growth_model_error <- function (logr, logc, data) {
  # Return sum-of-squares error between data growth points
  # and model initialized with parameters r and c.
  # Logs of r and c are passed to constraint the parameters
  # to positive values.

  ## Get times and log of data
  ts <- data[[1]]
  data_dyn <- log(data[[2]])
  model_dyn <- log(evaluate_growth_model(ts, logr, logc))

  ## DEBUG: plot to see if it makes sense
```

```r
  # plot(ts, data_dyn, type='p', col=3,
  #    main='Pa growth (model and data)',
  #    ylab="Pathogen load (fraction)",
  #    xlab="Time (hr)")
  # points(ts, model_dyn)

  ## Compute and return sum-of-squares error
  err <- sqrt(sum((data_dyn - model_dyn) ** 2))
  return(err)
}
```

Find errors for model instantiated to starting parameter values. Total error is the sum of the errors of the single cases.

```r
# Get error for model parameters to standard values
errA <- get_growth_model_error(log(0.08), log(0.0038210), paA_growth_norm)
errB <- get_growth_model_error(log(0.08), log(7e-04), paB_growth_norm)
errC <- get_growth_model_error(log(0.08), log(0.00019), paC_growth_norm)
total_err <- errA + errB + errC

errA
```

```
## [1] 0.5614984
```

```r
errB
```

```
## [1] 0.9288625
```

```r
errC
```

```
## [1] 1.593686
```

```r
total_err
```

```
## [1] 3.084047
```

Perform non-linear fit to find optimal parameters.

```r
minimize_me <- function (pars) {
  # Objective function.
  # Take colonization rates and growth rate.
  # Return total error between model and data-sets paX_growth_norm
  # CAREFUL: in-coded data-sets.
  logcA <- pars[[1]]
  logcB <- pars[[2]]
  logcC <- pars[[3]]
  logr <- pars[[4]]
  errA <- get_growth_model_error(logr, logcA, paA_growth_norm)
  errB <- get_growth_model_error(logr, logcB, paB_growth_norm)
  errC <- get_growth_model_error(logr, logcC, paC_growth_norm)
  total_err <- errA + errB + errC
  return(as.double(total_err))
}

ans <- optim(par=c(log(0.0038210), log(7e-04), log(0.00019), log(0.08)),
             fn=minimize_me,
             method = "Nelder-Mead")

ans
```

```
## $par
## [1] -5.604069 -7.077498 -7.999112 -2.471371
##
## $value
## [1] 2.512691
##
## $counts
## function gradient
##      219       NA
##
## $convergence
## [1] 0
##
## $message
## NULL
```

```
exp(ans$par)
```

```
## [1] 0.0036828467 0.0008438818 0.0003357606 0.0844689926
```

Show fit with parameters found by minizing objective function.

```
library("pracma")

logcA <- ans$par[[1]]
logcB <- ans$par[[2]]
logcC <- ans$par[[3]]
logr <- ans$par[[4]]

ts <- linspace(1, 120, n=100)
paA_model_dyn <- evaluate_growth_model(ts, logr, logcA)
paB_model_dyn <- evaluate_growth_model(ts, logr, logcB)
paC_model_dyn <- evaluate_growth_model(ts, logr, logcC)

plot(ts, paA_model_dyn, type='l', col=2,
     main='Pa growth (model and data)',
     log='y', ylim=c(10**-4, 1),
     ylab="Pathogen load (fraction)",
     xlab="Time (hr)")
points(ts, paB_model_dyn, col=3, type = 'l')
points(ts, paC_model_dyn, col=4, type = 'l')

points(paA_growth_norm[[1]], paA_growth_norm[[2]], col=2, type = 'p')
points(paB_growth_norm[[1]], paB_growth_norm[[2]], col=3, type = 'p')
points(paC_growth_norm[[1]], paC_growth_norm[[2]], col=4, type = 'p')
```

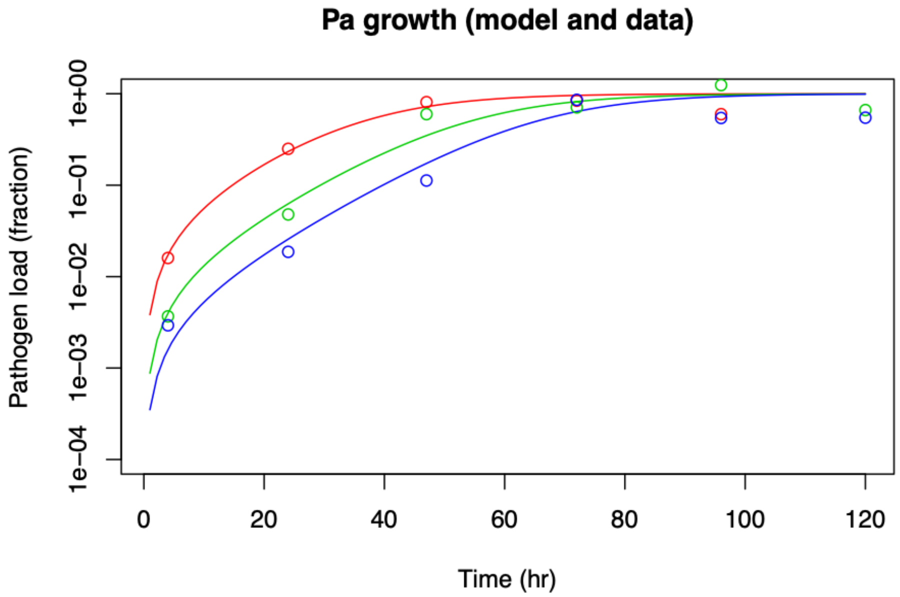

Find optmial parameter values to be reported in Supplementary Table 1.

```
as.double(exp(logr))  # Pa growth rate
```

```
## [1] 0.08446899
```

```
as.integer(exp(logcA) * KPa)  # Pa colonization rate for pre-incub 48 hr
```

```
## [1] 1031
```

```
as.integer(exp(logcB) * KPa)  # Pa colonization rate for pre-incub 24 hr
```

```
## [1] 236
```

```
as.integer(exp(logcC) * KPa)  # Pa colonization rate for pre-incub 4 hr
```

```
## [1] 94
```

Find error by bootstrapping to *Pa* growth curves in Fig. 4-C

Re-sample growth curve by bootstrapping.

```
resample_norm_growth_curve <- function (ds_growth, k) {
  # MC bootstrap raw growth data and return
  # normalized mean curve from resampled
  resampled_pts <- double()
  for (i in 1:nrow(ds_growth)) {
    vec <- as.vector(ds_growth[i, 2:5])  # Pick dataset at time point
    vec <- vec[!is.na(vec)]  # remove NaNs
    data <- sample(vec, size = length(vec), replace = T) # Resample
    m <- mean(data) / KPa # Compute statistics for resampled set
    resampled_pts <- c(resampled_pts, m)
  }
```

```
  ts <- as.vector(ds_growth[[1]])
  return(list(ts, resampled_pts))
}
```

Show bootstrapped curves.

```
paA_growth_norm_inst <- resample_norm_growth_curve(paA_growth, KPa)
plot(paA_growth_norm_inst[[1]], paA_growth_norm_inst[[2]],
     log='y',
     ylim=c(10**-2, 1.5),
     xlab='Time (hr)',
     ylab='Pathogen load (fraction)',
     main='Various resampling of Pa 48h growth curve')

for (i in 1:30) {
  paA_growth_norm_inst <- resample_norm_growth_curve(paA_growth, KPa)
  points(paA_growth_norm_inst[[1]], paA_growth_norm_inst[[2]], col=i)
}
```

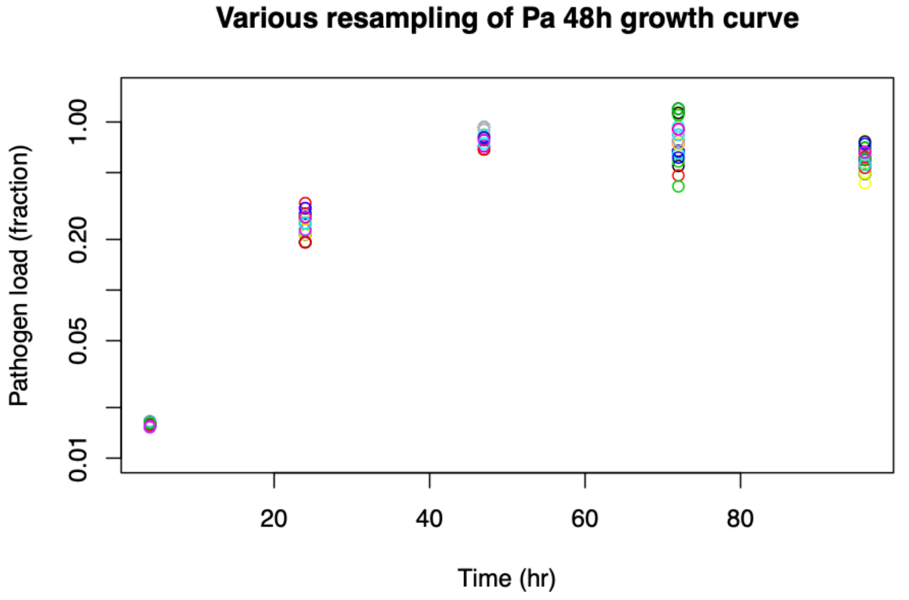

Bootstrap growth curves. Fit model to bootstrapped curves.

```
find_par_instance <- function () {
  # Bootstrap growth curves and fit model to curves.
  # Return parameter instance.

  paA_growth_norm_inst <- resample_norm_growth_curve(paA_growth, KPa)
  paB_growth_norm_inst <- resample_norm_growth_curve(paB_growth, KPa)
  paC_growth_norm_inst <- resample_norm_growth_curve(paC_growth, KPa)

  minimize_me2 <- function (pars) {
    # v2: works on resampled growth curve.
```

```
    # Objective function.
    # Take colonization rates and growth rate.
    # Return total error between model and data-sets paX_growth_norm
    # CAREFUL: in-coded data-sets.
    logcA <- pars[[1]]
    logcB <- pars[[2]]
    logcC <- pars[[3]]
    logr <- pars[[4]]
    errA <- get_growth_model_error(logr, logcA, paA_growth_norm_inst)
    errB <- get_growth_model_error(logr, logcB, paB_growth_norm_inst)
    errC <- get_growth_model_error(logr, logcC, paC_growth_norm_inst)
    total_err <- errA + errB + errC
    return(as.double(total_err))
  }

  ans <- optim(par=c(log(0.0038210), log(7e-04), log(0.00019), log(0.08)),
        fn=minimize_me2,
        method = "Nelder-Mead")

  # print(exp(ans$par))
  return(ans)
}
```

```
show_histograms <- function() {
  ## Generate distributions
  cAs <- vector()
  cBs <- vector()
  cCs <- vector()
  rs <- vector()
  for (i in 1:500) {
    ans <- find_par_instance()
    cAs <- c(cAs, exp(ans$par[[1]]))
    cBs <- c(cBs, exp(ans$par[[2]]))
    cCs <- c(cCs, exp(ans$par[[3]]))
    rs <- c(rs, exp(ans$par[[4]]))
  }
  ## Compute s.e.m.
  r_sem <- round(sd(rs), digits = 5)
  cA_sem <- round(sd(cAs), digits = 5)
  cB_sem <- round(sd(cBs), digits = 5)
  cC_sem <- round(sd(cCs), digits = 5)

  ## Compute mean
  r_val <- round(mean(rs), digits = 5)
  cA_val <- round(mean(cAs), digits = 5)
  cB_val <- round(mean(cBs), digits = 5)
  cC_val <- round(mean(cCs), digits = 5)

  ## Plot histograms
  hist(rs, main=paste('Pa growth rate. val:', r_val, "+/-", r_sem))
  hist(cAs, main=paste('Pa col. rate. val:', cA_val, "+/-", cA_sem))
  hist(cBs, main=paste('Pa col. rate. val:', cB_val, "+/-", cB_sem))
  hist(cCs, main=paste('Pa col. rate. val:', cC_val, "+/-", cC_sem))
```

```
}
show_histograms()
```

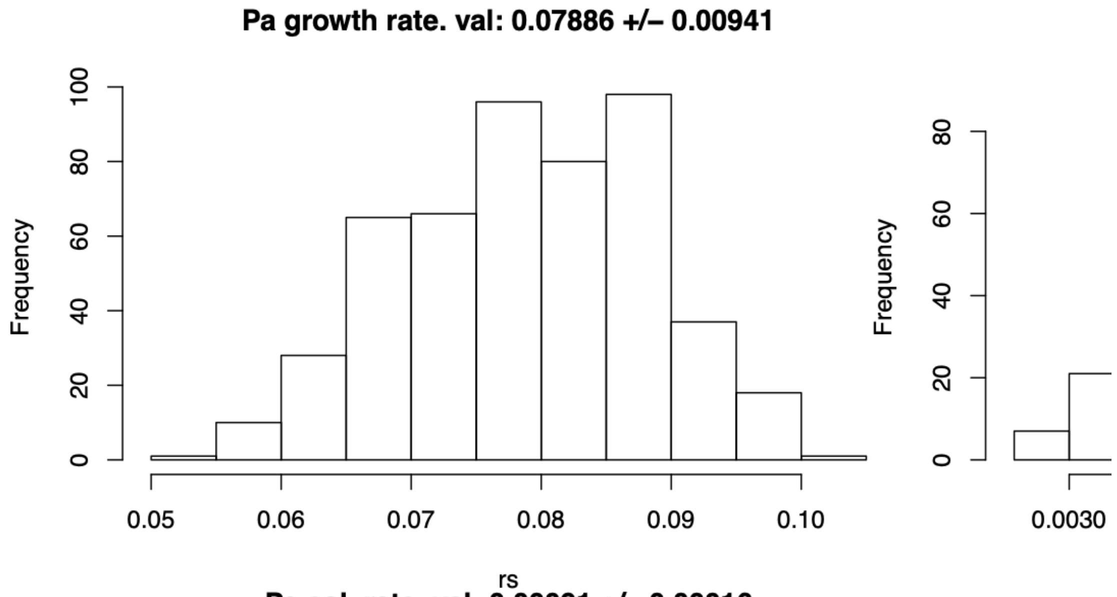

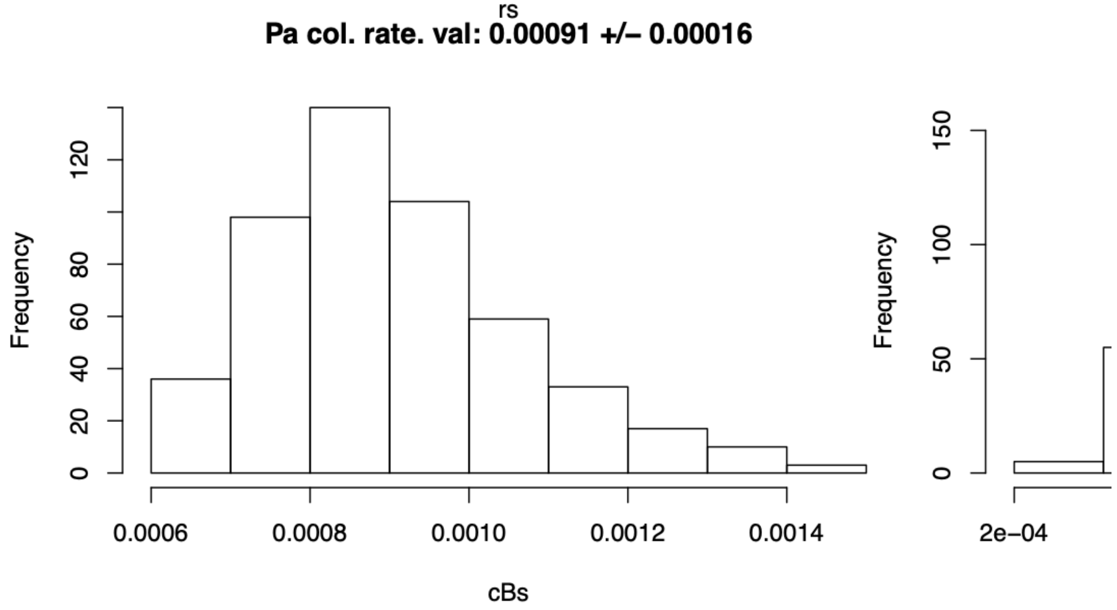

Colonization rate errors are normalized by carrying capacities. Reported in Supplementary Table 1.

```
0.00043* KPa
```

```
## [1] 120.4
```

```
0.00017 * KPa
```

```
## [1] 47.6
```

```
5e-05 * KPa
```

```
## [1] 14
```

Non-linear fit of *Pa*, *Sm* and *Se* growth/survival curves in Fig. 3

Fit model to mean curves (survival and growth curves)

Load growth curves from CSV files

```
options <- list(
            header = FALSE,
            col.names =  c("times hr","Cells replica 1","Cells replica 2","Cells replica 3","Cells repl
            )

args <- c(paste(wd1, 'pa_growth.csv', sep = ""), options)
pa_growth <- do.call(read.csv, args)

args <- c(paste(wd1, 'sm_growth.csv', sep = ""), options)
sm_growth <- do.call(read.csv, args)

args <- c(paste(wd1, 'se_growth.csv', sep = ""), options)
se_growth <- do.call(read.csv, args)
```

Display growth data and mean curve of pathogen loads. Draw horizontal line to inspect carrying capacity.

Display growth data, find carrying capacity by inspection and return normalized growth curves.

```
display_growth_data <- function (dfs, title_text, K) {
  # Print growth data from `dfs` along with mean curve.
  # Title figure `title_text`.
  # Draw horizontal line with intercept `K`
  # Return normalized mean curve as 2d list (times, vals).

  ## Get data as matrices
  cells <- as.matrix(dfs[, 2:5])
  ts <- matrix(rep(dfs[[1]], 4), nrow = 4, byrow = TRUE)
  ts <- t(ts)

  ## Compute mean curve
  mean_curve_cells <- rowMeans(cells, na.rm = TRUE)
  mean_curve_ts <- ts[,1]

  ## Flatten data for plotting
  ts <- as.vector(ts)
  cells <- as.vector(cells)

  ## Plot raw data
  plot(ts, cells,
```

```
        xlim = c(0, 180),
        ylim = c(10**2, 2*10**6),
        log = "y",
        main = title_text,
        xlab = 'Time (hr)',
        ylab = 'Pathogen load (cells)'
        )

  ## Add mean curve to plot
  points(mean_curve_ts, mean_curve_cells, type = 'l')

  ## Add carrying capacity line
  abline(h = K, lt=2, col='red')

  return(list(mean_curve_ts, mean_curve_cells / K))
}

KPa <- 2.8 * 10**5
KSm <- 1.1 * 10**5
KSe <- 1.7 * 10**6
pa_growth_norm <- display_growth_data(pa_growth, "Pa", KPa)
```

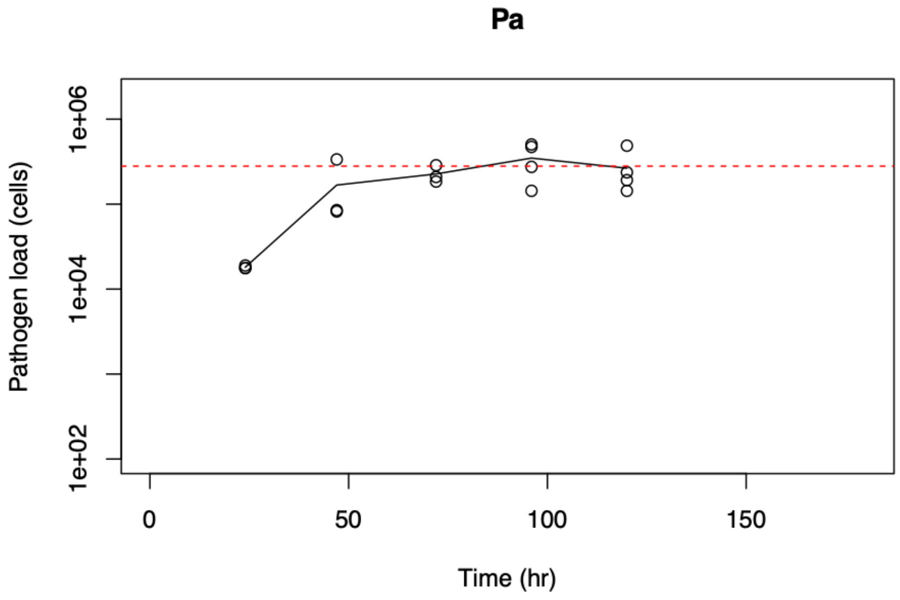


```
sm_growth_norm <- display_growth_data(sm_growth, "Sm", KSm)
```

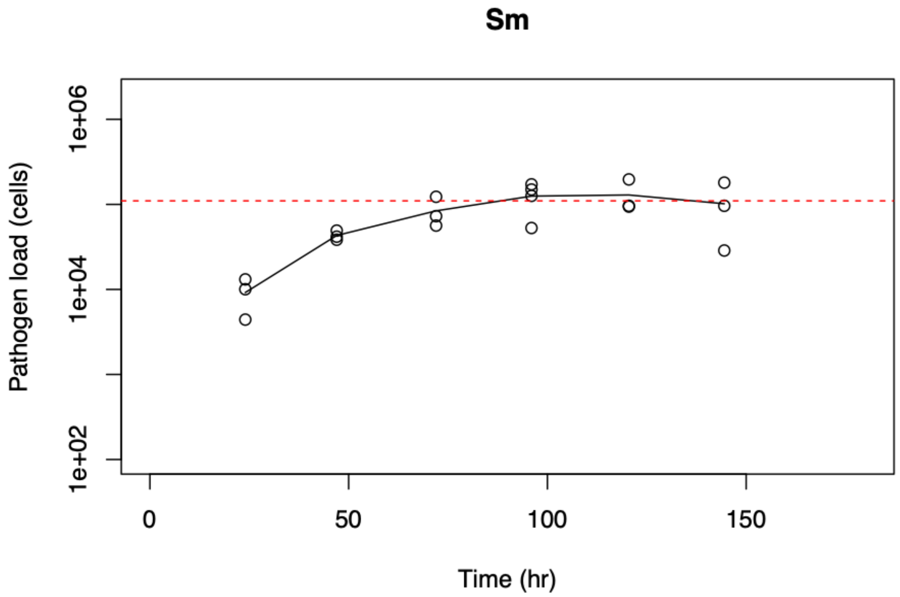


```
se_growth_norm <- display_growth_data(se_growth, "Se", KSe)
```

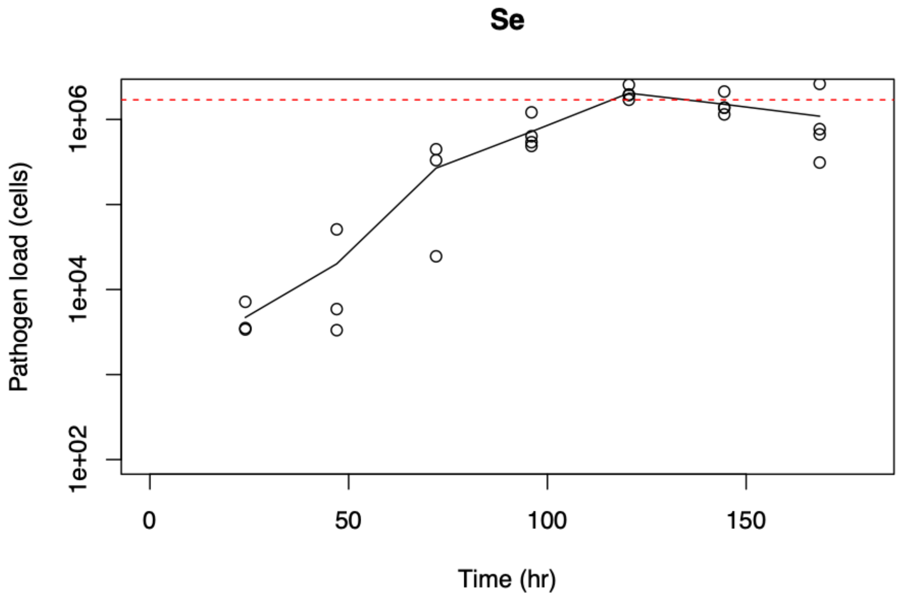

Display survival curve data and get mean curve.

```
display_surv_data <- function (dfs, title_text) {
  # Display survival data and mean curve
```

```
  ## Get mean survival curve
  w_rows <- list()
  for (df in dfs) {
    w_rows <- as.double(c(w_rows, df[[2]]))
  }
  row_matrix <- matrix(w_rows, nrow = length(dfs), byrow = TRUE)
  mean_sc <- colMeans(row_matrix)

  ## Plot mean surv curve
  times <- dfs[[1]][[1]]
  plot(
      times, mean_sc,
      ylab='Fraction of worms surviving',
      xlab='Time (hr)',
      log="y",
      xlim=c(0, 180),
      ylim=c(10**-3, 1.2),
      main=title_text,
      type='l'
    )

  ## Add raw data
  for (i in 1:length(dfs)) {
    df <- dfs[[i]]
    ts <- df[[1]]
    pts <- df[[2]]
    points(ts, pts, col=i, type='p')
  }

   ## Return mean surv curve
  return(list(times, mean_sc))
}

pa_surv <- display_surv_data(pa_dfs, "Pa surv. data")
```

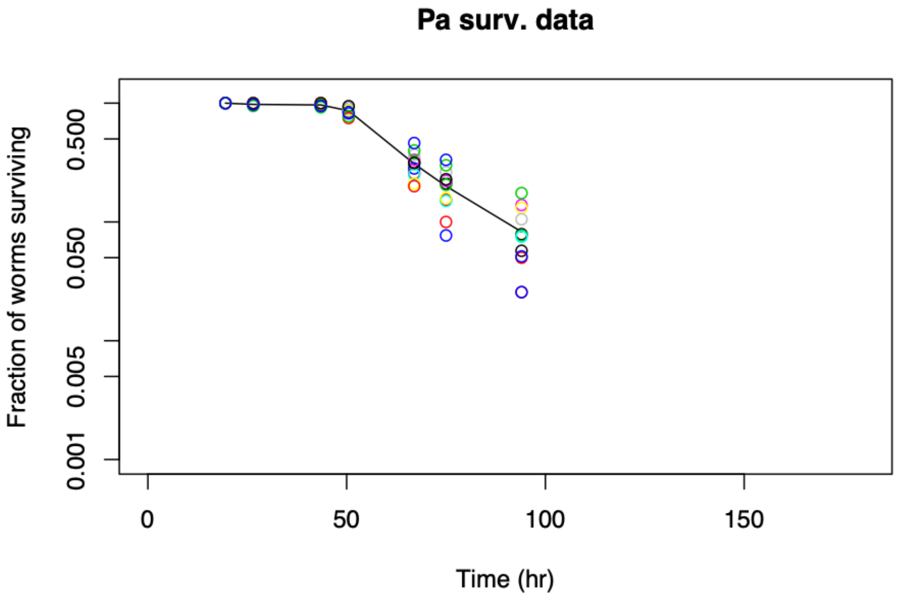


```
sm_surv <- display_surv_data(sm_dfs, "Sm surv. data")
```

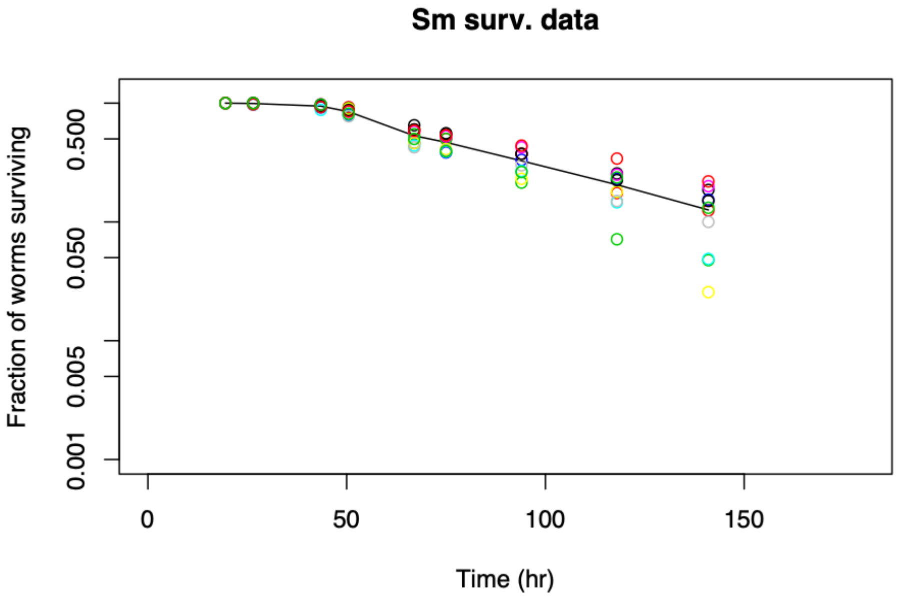


```
se_surv <- display_surv_data(se_dfs, "Se surv. data")
```

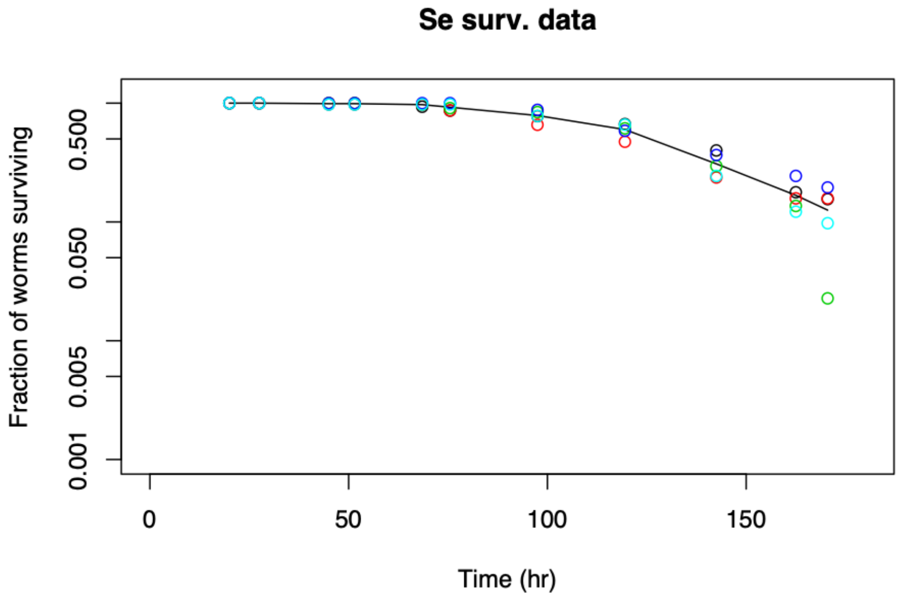

Compute pathogen load model solution.

```
evaluate_surv_model <- function (t, logr, logc, logd) {
  # Return survival function at time t.
  # `t` can be a list of times.
  # log transform r, c and delta so that optimization problem is unconstrained.
  ## ADD CARRYING CAPACITY K

  library("Brobdingnag")  # Handles very large number

  ## Multiplies whole exponential
  coeff2 <- as.brob(exp(logc - logr + logd))
  coeff <- exp(coeff2 * t)

  num <- as.brob(exp(logc) + exp(logr))
  den <- exp(logc + num*t) + exp(logr)

  frac <- (num/den) ** exp(logd-logr)

  ans <- as.double(frac * coeff)
  return (ans)
}
```

Show model predictions on data for starting parameter values.

Display growth and survival data with models predictions initialized to starting parameter values.

```
## Show data and model prediction for Pa
ts <- pa_surv[[1]]
pts <- pa_surv[[2]]
plot(ts, pts,
```

```
     log="y",
     col='dark green',
     main='Pa surv data',
     ylim=c(10**-4, 1),
     xlim=c(0, 180)
     )
ts <- linspace(1, 180, n=100)
pa_model_surv_dyn <- evaluate_surv_model(ts, log(0.09), log(0.001), log(0.058))
pa_model_growth_dyn <- evaluate_growth_model(ts, log(0.09), log(0.001))
points(ts, pa_model_surv_dyn, type='l', col='dark green')
points(pa_growth_norm[[1]], pa_growth_norm[[2]], type='p', pch=2, col='dark green')
points(ts, pa_model_growth_dyn, type='l', lty=2, col='dark green')
```

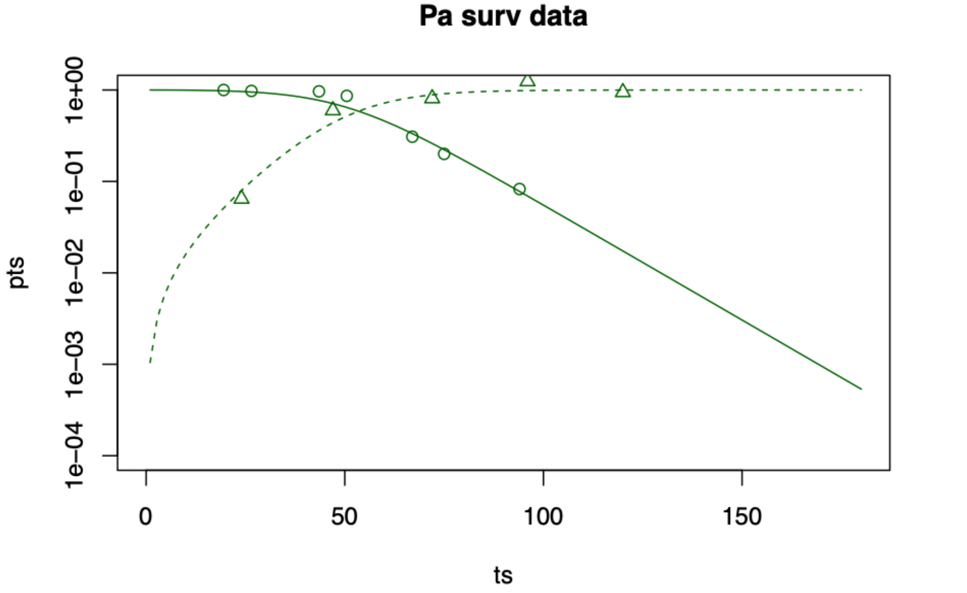


```
## Show data and model prediction for Sm
ts <- sm_surv[[1]]
pts <- sm_surv[[2]]
plot(ts, pts,
     log="y",
     col='brown',
     main='Sm surv data',
     ylim=c(10**-4, 1),
     xlim=c(0, 180)
     )
ts <- linspace(1, 180, n=100)
sm_model_surv_dyn <- evaluate_surv_model(ts, log(0.07), log(0.002), log(0.02))
sm_model_growth_dyn <- evaluate_growth_model(ts, log(0.09), log(0.001))
points(ts, sm_model_surv_dyn, type='l', col='brown')
points(sm_growth_norm[[1]], sm_growth_norm[[2]], type='p', pch=2, col='brown')
points(ts, sm_model_growth_dyn, type='l', lty=2, col='brown')
```

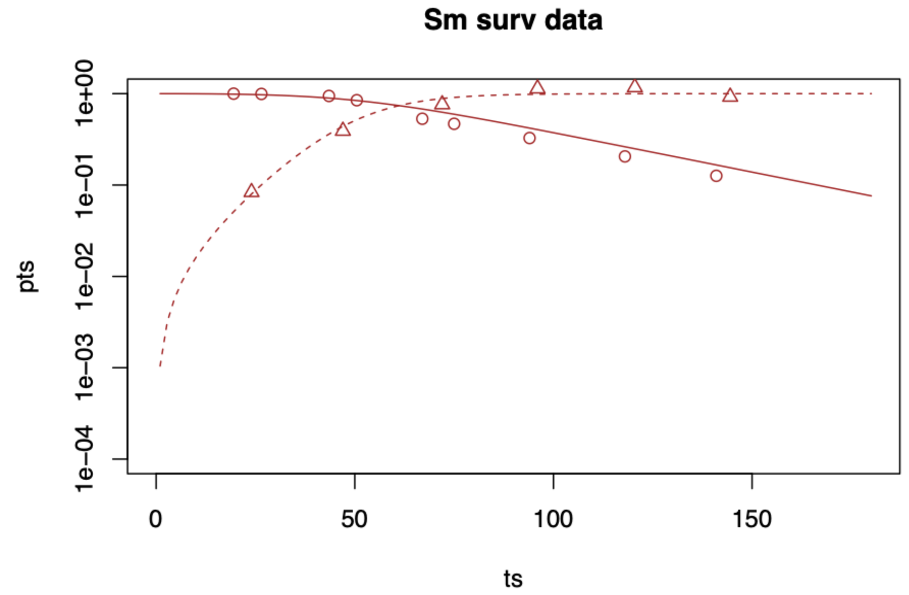


```
## Show data and model prediction for Se
ts <- se_surv[[1]]
pts <- se_surv[[2]]
plot(ts, pts,
     log="y",
     col='purple',
     main='Se surv data',
     ylim=c(10**-4, 1),
     xlim=c(0, 180)
     )
ts <- linspace(1, 180, n=100)
se_model_surv_dyn <- evaluate_surv_model(ts, log(0.08), log(0.00003), log(0.033))
se_model_growth_dyn <- evaluate_growth_model(ts, log(0.09), log(0.00003))
points(ts, se_model_surv_dyn, type='l', col='purple')
points(se_growth_norm[[1]], se_growth_norm[[2]], type='p', pch=2, col='purple')
points(ts, se_model_growth_dyn, type='l', lty=2, col='purple')
```

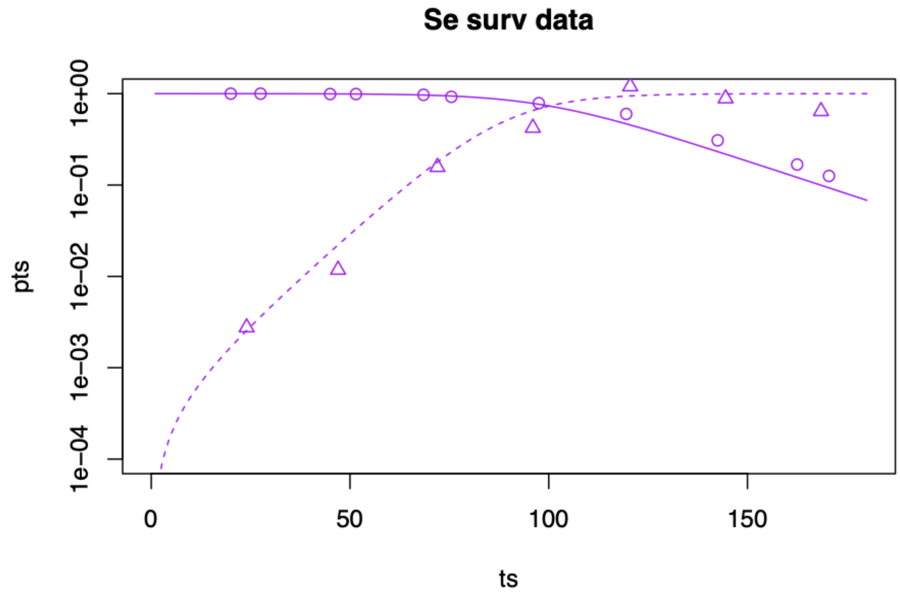

Compute sum-of-squares error between growth model solution and (a single) data set.

```
get_growth_model_error <- function (logr, logc, data) {
  # Return sum-of-squares error between data growth points
  # and model initialized with parameters r and c.
  # Logs of r and c are passed to constraint the parameters
  # to positive values.

  ## Get times and log of data
  ts <- data[[1]]
  data_dyn <- log(data[[2]])
  model_dyn <- log(evaluate_growth_model(ts, logr, logc))

  # # DEBUG: plot to see if it makes sense
  # plot(ts, data_dyn, type='p', col=3,
  #    main='Pa growth (model and data)',
  #    ylab="Pathogen load (fraction)",
  #    xlab="Time (hr)")
  # points(ts, model_dyn)

  ## Compute and return sum-of-squares error
  err <- sqrt(sum((data_dyn - model_dyn) ** 2))
  return(err)
}
```

Compute sum-of-squares error between survival model solution and (a single data) set.

```
get_surv_model_error <- function (logr, logc, logd, data) {
  # Return sum-of-squares error between data surv points
  # and model initialized with parameters r, c and delta.
  # Logs of pars are passed to constraint the parameters
```

```r
  # to positive values.

  ## Get times and log of data
  ts <- data[[1]]
  data_dyn <- log(data[[2]])
  model_dyn <- log(evaluate_surv_model(ts, logr, logc, logd))

  # # DEBUG: plot to see if it makes sense
  # plot(ts, data_dyn, type='p', col=3,
  #     xlab="Time (hr)")
  # points(ts, model_dyn)

  ## Compute and return sum-of-squares error
  err <- sqrt(sum((data_dyn - model_dyn) ** 2))
  return(err)
}
```

Object functions for the three datasets.

```r
pa_minimize_me <- function(pars) {
  logr <- pars[[1]]
  logc <- pars[[2]]
  logd <- pars[[3]]
  err_surv <- get_surv_model_error(logr, logc, logd, pa_surv)
  err_growth <- get_growth_model_error(logr, logc, pa_growth_norm)
  tot_err <- err_growth + err_surv
  return(tot_err)
}

sm_minimize_me <- function(pars) {
  logr <- pars[[1]]
  logc <- pars[[2]]
  logd <- pars[[3]]
  err_surv <- get_surv_model_error(logr, logc, logd, sm_surv)
  err_growth <- get_growth_model_error(logr, logc, sm_growth_norm)
  tot_err <- err_growth + err_surv
  return(tot_err)
}

se_minimize_me <- function(pars) {
  logr <- pars[[1]]
  logc <- pars[[2]]
  logd <- pars[[3]]
  err_surv <- get_surv_model_error(logr, logc, logd, se_surv)
  err_growth <- get_growth_model_error(logr, logc, se_growth_norm)
  tot_err <- err_growth + err_surv
  return(tot_err)
}
```

Find *Pa* optimal parameters.

```r
pa_ans <- optim(par=c(log(0.09), log(0.001), log(0.058)),
             fn=pa_minimize_me,
             method = "Nelder-Mead")
```

```r
pa_ans
```

```
## $par
## [1] -2.069538 -7.765565 -2.976791
##
## $value
## [1] 0.6593284
##
## $counts
## function gradient
##      154       NA
##
## $convergence
## [1] 0
##
## $message
## NULL
```

```r
exp(pa_ans$par)
```

```
## [1] 0.12624408 0.00042409 0.05095611
```

Find *Sm* optimal parameters.

```r
sm_ans <- optim(par=c(log(0.07), log(0.002), log(0.02)),
             fn=sm_minimize_me,
             method = "Nelder-Mead")

sm_ans
```

```
## $par
## [1] -2.500719 -6.720666 -3.719750
##
## $value
## [1] 0.5518004
##
## $counts
## function gradient
##      160       NA
##
## $convergence
## [1] 0
##
## $message
## NULL
```

```r
exp(sm_ans$par)
```

```
## [1] 0.082026001 0.001205735 0.024240024
```

Find *Se* optimal parameters.

```r
se_ans <- optim(par=c(log(0.08), log(0.00003), log(0.033)),
             fn=se_minimize_me,
             method = "Nelder-Mead")

se_ans
```

```
## $par
## [1]  -2.537121 -10.314167  -3.585918
##
## $value
## [1] 0.9313928
##
## $counts
## function gradient
##       98       NA
##
## $convergence
## [1] 0
##
## $message
## NULL
```

```
exp(se_ans$par)
```

```
## [1] 7.909376e-02 3.315999e-05 2.771121e-02
```

Show data and best model predictions for optimal parameters.

```
## Show data and model prediction for Pa
logr <- pa_ans$par[[1]]
logc <- pa_ans$par[[2]]
logd <- pa_ans$par[[3]]
ts <- pa_surv[[1]]
pts <- pa_surv[[2]]
plot(ts, pts,
     log="y",
     col='dark green',
     main='Pa surv data',
     ylim=c(10**-4, 1),
     xlim=c(0, 180)
     )
ts <- linspace(1, 180, n=100)
pa_model_surv_dyn <- evaluate_surv_model(ts, logr, logc, logd)
pa_model_growth_dyn <- evaluate_growth_model(ts, logr, logc)
points(ts, pa_model_surv_dyn, type='l', col='dark green')
points(pa_growth_norm[[1]], pa_growth_norm[[2]], type='p', pch=2, col='dark green')
points(ts, pa_model_growth_dyn, type='l', lty=2, col='dark green')
```

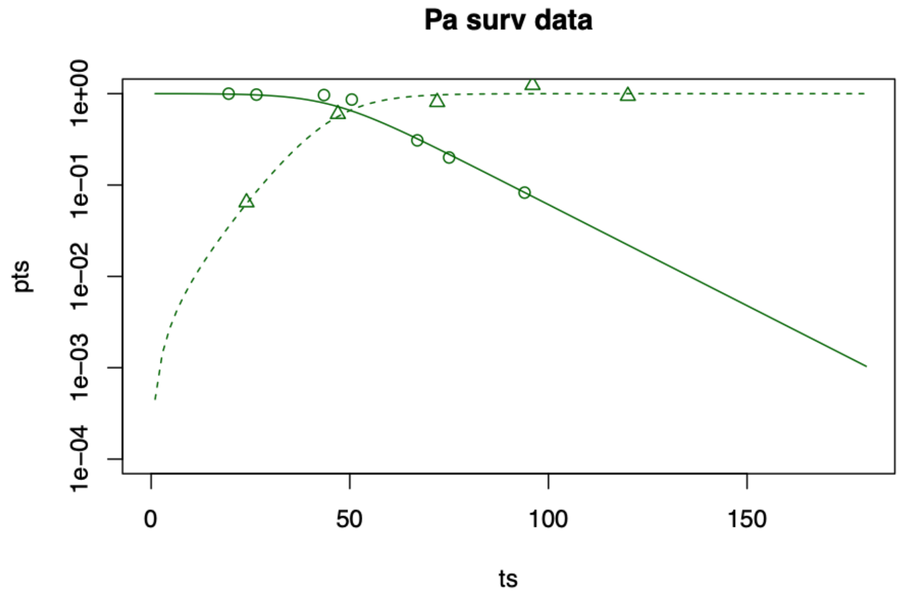


```
## Show data and model prediction for Sm
logr <- sm_ans$par[[1]]
logc <- sm_ans$par[[2]]
logd <- sm_ans$par[[3]]
ts <- sm_surv[[1]]
pts <- sm_surv[[2]]
plot(ts, pts,
     log="y",
     col='brown',
     main='Sm surv data',
     ylim=c(10**-4, 1),
     xlim=c(0, 180)
     )
ts <- linspace(1, 180, n=100)
sm_model_surv_dyn <- evaluate_surv_model(ts, logr, logc, logd)
sm_model_growth_dyn <- evaluate_growth_model(ts, logr, logc)
points(ts, sm_model_surv_dyn, type='l', col='brown')
points(sm_growth_norm[[1]], sm_growth_norm[[2]], type='p', pch=2, col='brown')
points(ts, sm_model_growth_dyn, type='l', lty=2, col='brown')
```

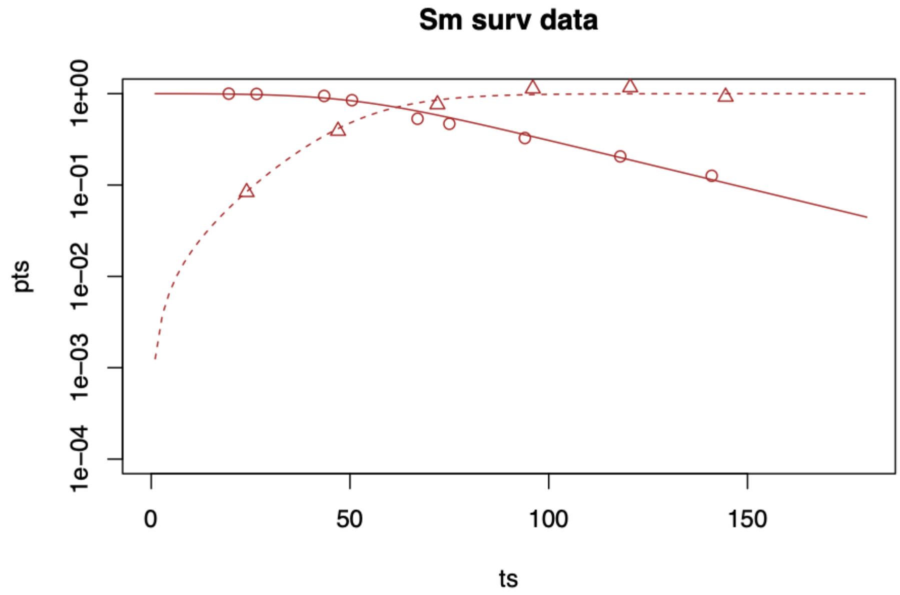


```
## Show data and model prediction for Se
logr <- se_ans$par[[1]]
logc <- se_ans$par[[2]]
logd <- se_ans$par[[3]]
ts <- se_surv[[1]]
pts <- se_surv[[2]]
plot(ts, pts,
     log="y",
     col='purple',
     main='Se surv data',
     ylim=c(10**-4, 1),
     xlim=c(0, 180)
     )
ts <- linspace(1, 180, n=100)
se_model_surv_dyn <- evaluate_surv_model(ts, logr, logc, logd)
se_model_growth_dyn <- evaluate_growth_model(ts, logr, logc)
points(ts, se_model_surv_dyn, type='l', col='purple')
points(se_growth_norm[[1]], se_growth_norm[[2]], type='p', pch=2, col='purple')
points(ts, se_model_growth_dyn, type='l', lty=2, col='purple')
```

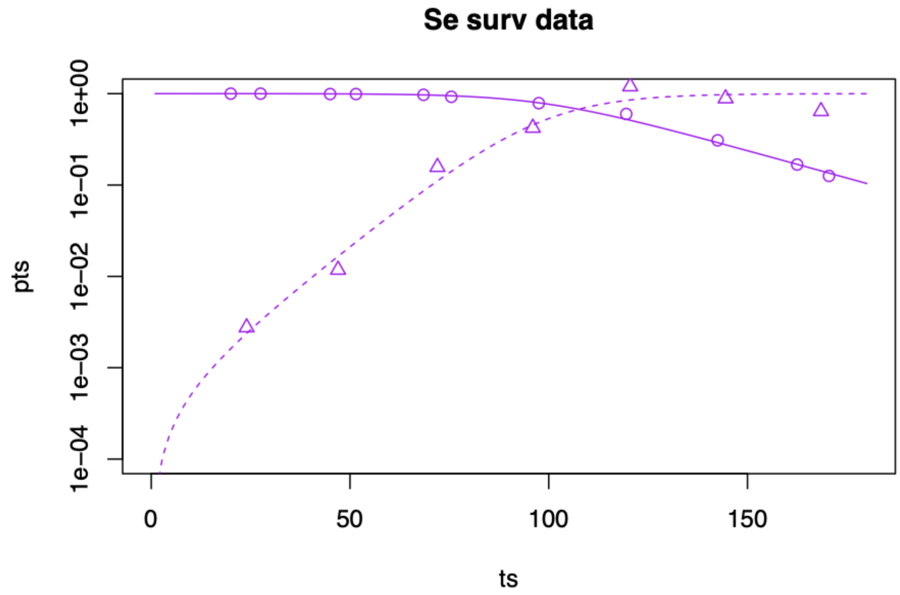

Print parameter values to be used in paper.

```
rPa <- exp(pa_ans$par[[1]])
cPa <- exp(pa_ans$par[[2]])
dPa <- exp(pa_ans$par[[3]])
c(rPa, cPa * KPa, dPa)
```

```
## [1]   0.12624408 118.74521047   0.05095611
```

```
rSm <- exp(sm_ans$par[[1]])
cSm <- exp(sm_ans$par[[2]])
dSm <- exp(sm_ans$par[[3]])
c(rSm, cSm * KSm, dSm)
```

```
## [1]   0.08202600 132.63090127   0.02424002
```

```
rSe <- exp(se_ans$par[[1]])
cSe <- exp(se_ans$par[[2]])
dSe <- exp(se_ans$par[[3]])
c(rSe, cSe * KSe, dSe)
```

```
## [1]  0.07909376 56.37198092  0.02771121
```

Find errors by bootstrapping

Re-sample growth curve by bootstrapping.

```
resample_norm_growth_curve <- function (ds_growth, K) {
  # MC bootstrap raw growth data and return
  # normalized mean curve from resampled
  resampled_pts <- double()
  for (i in 1:nrow(ds_growth)) {
    vec <- as.vector(ds_growth[i, 2:5])  # Pick dataset at time point
```

```
    vec <- vec[!is.na(vec)]  # remove NaNs
    data <- sample(vec, size = length(vec), replace = T) # Resample
    m <- mean(data) / K # Compute statistics for resampled set
    resampled_pts <- c(resampled_pts, m)
  }
  ts <- as.vector(ds_growth[[1]])
  return(list(ts, resampled_pts))
}
```

Show bootstrapped growth curves.

```
pa_growth_norm_inst <- resample_norm_growth_curve(pa_growth, KPa)
plot(pa_growth_norm_inst[[1]], pa_growth_norm_inst[[2]],
     log='y',
     ylim=c(10**-2, 1.5),
     xlab='Time (hr)',
     ylab='Pathogen load (fraction)',
     main='Resampling Pa growth curve')

for (i in 1:30) {
  pa_growth_norm_inst <- resample_norm_growth_curve(pa_growth, KPa)
  points(pa_growth_norm_inst[[1]], pa_growth_norm_inst[[2]], col=i)
}
```

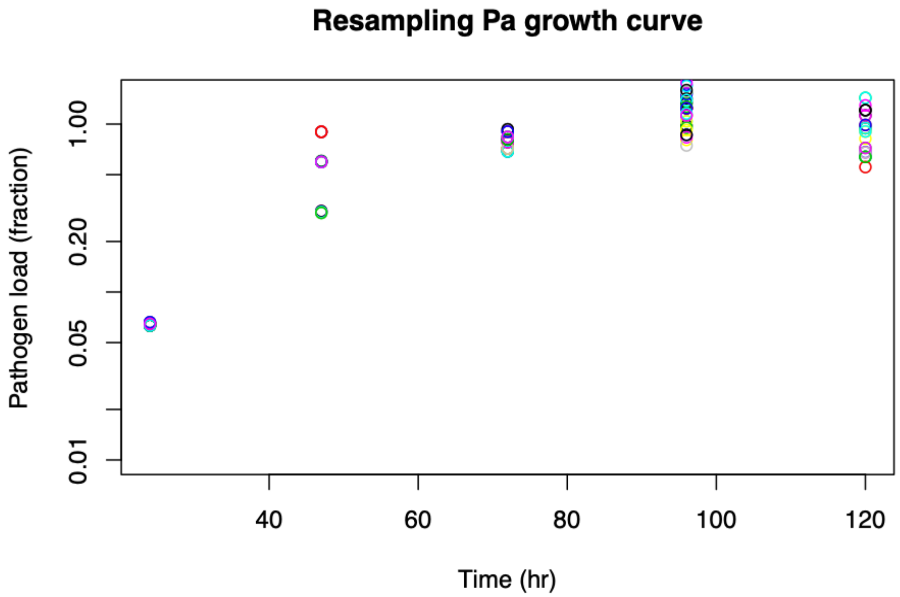


```
sm_growth_norm_inst <- resample_norm_growth_curve(sm_growth, KSm)
plot(sm_growth_norm_inst[[1]], sm_growth_norm_inst[[2]],
     log='y',
     ylim=c(10**-2, 1.5),
     xlab='Time (hr)',
     ylab='Pathogen load (fraction)',
```

```
     main='Resampling Sm growth curve')

for (i in 1:30) {
  sm_growth_norm_inst <- resample_norm_growth_curve(sm_growth, KSm)
  points(sm_growth_norm_inst[[1]], sm_growth_norm_inst[[2]], col=i)
}
```

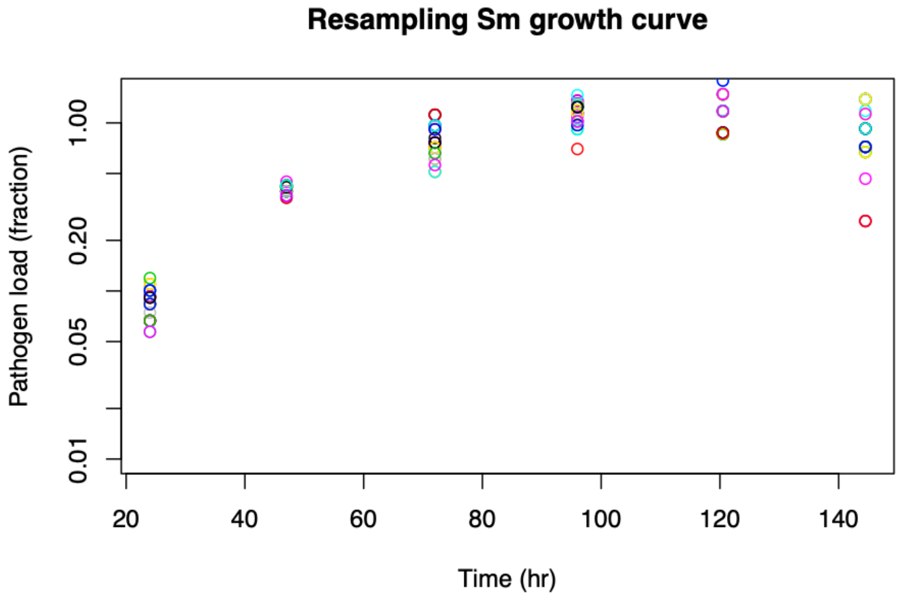


```
se_growth_norm_inst <- resample_norm_growth_curve(se_growth, KSe)
plot(se_growth_norm_inst[[1]], se_growth_norm_inst[[2]],
     log='y',
     ylim=c(10**-2, 1.5),
     xlab='Time (hr)',
     ylab='Pathogen load (fraction)',
     main='Resampling of Se growth curve')

for (i in 1:30) {
  se_growth_norm_inst <- resample_norm_growth_curve(se_growth, KSe)
  points(se_growth_norm_inst[[1]], se_growth_norm_inst[[2]], col=i)
}
```

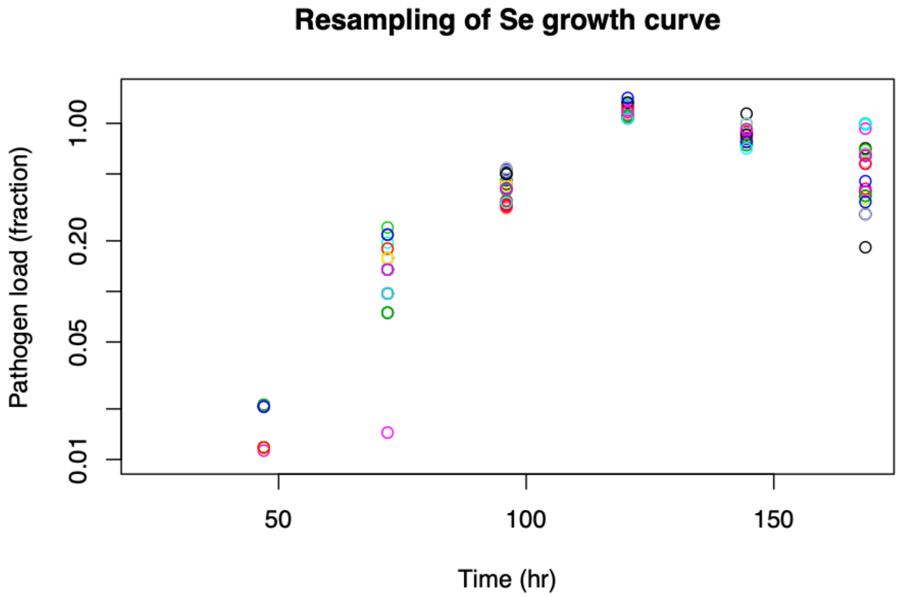

Re-sample survival curve by bootstrapping.

```
resample_surv_curve <- function (ds_surv) {
  # MC bootstrap from dfs of survival curves
  ## Get times
  ts <- ds_surv[[1]][[1]]
  n_pts <- length(ts)
  ## Arrange all data in matrix
  a <- matrix(data = NaN, ncol = n_pts)  # Create pseudo-empty matrix
  for (df in ds_surv) {
    a <- rbind(a, df[[2]])  # append dataset
  }
  a <- a[-1,]  # remove NaN row

  ## Re-sample dataset
  sampled <- apply(a, MARGIN=2, FUN=sample, size=1)
  # plot(ts, sampled, log="y")
  ## Return instnace
  return(list(ts, sampled))
}
```

Show resampled survival curves.

```
## Resample Pa surv curves
plot(NULL,
     log="y",
     xlim=c(0, 120),
     ylim=c(10**-2, 1.2),
     xlab="Time (hr)",
     ylab="Fraction of worms surviving",
     main="Pa resampled surv"
```

```
     )
for (i in 1:100) {
  ans <- resample_surv_curve(pa_dfs)
  ts <- ans[[1]]
  pts <- ans[[2]]
  points(ts, pts, col=i)
}
```

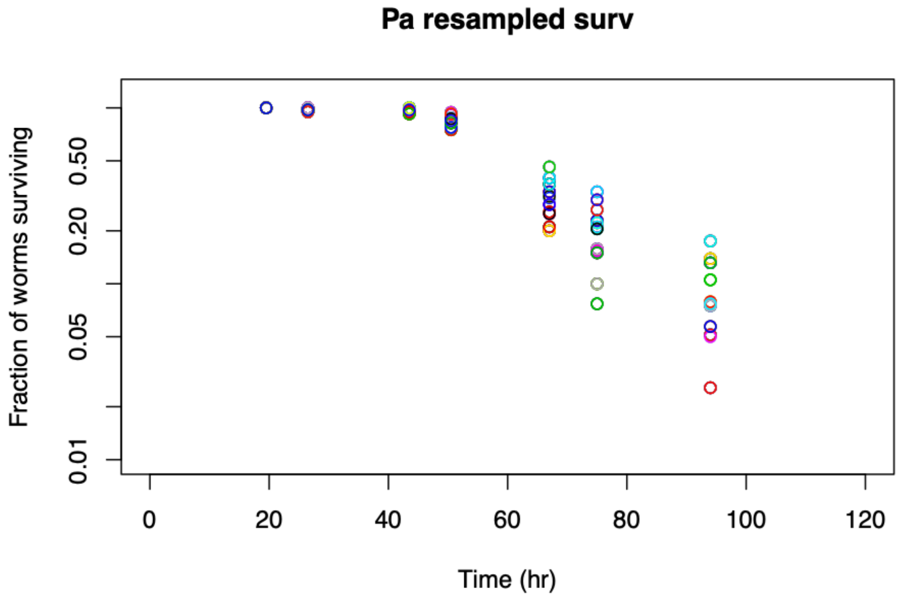


```
## Resample Sm surv curves
plot(NULL,
     log="y",
     xlim=c(0, 160),
     ylim=c(10**-2, 1.2),
     xlab="Time (hr)",
     ylab="Fraction of worms surviving",
     main="Sm resampled surv"
     )
for (i in 1:100) {
  ans <- resample_surv_curve(sm_dfs)
  ts <- ans[[1]]
  pts <- ans[[2]]
  points(ts, pts, col=i)
}
```

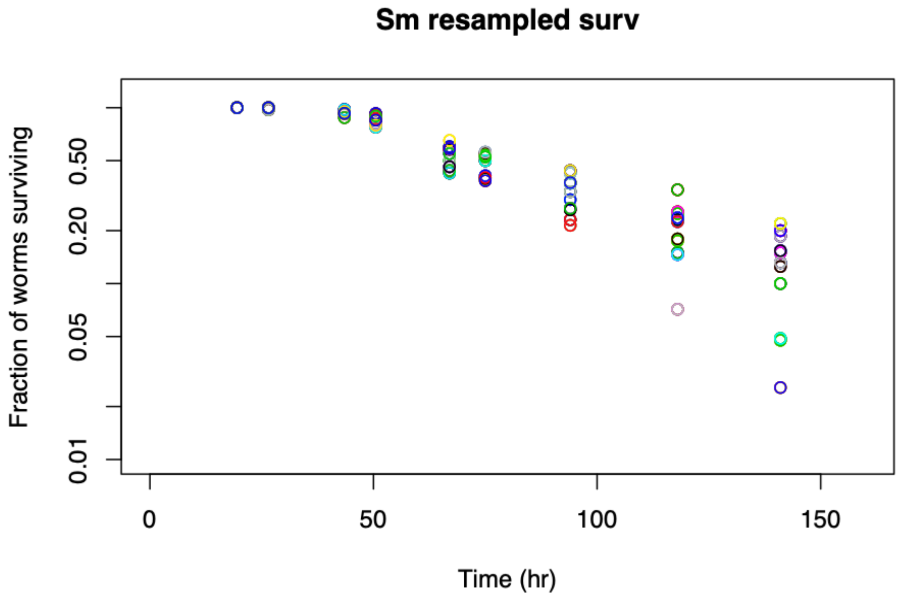


```
## Resample Se surv curves
plot(NULL,
     log="y",
     xlim=c(0, 180),
     ylim=c(10**-2, 1.2),
     xlab="Time (hr)",
     ylab="Fraction of worms surviving",
     main="Se resampled surv"
     )
for (i in 1:100) {
  ans <- resample_surv_curve(se_dfs)
  ts <- ans[[1]]
  pts <- ans[[2]]
  points(ts, pts, col=i)
}
```

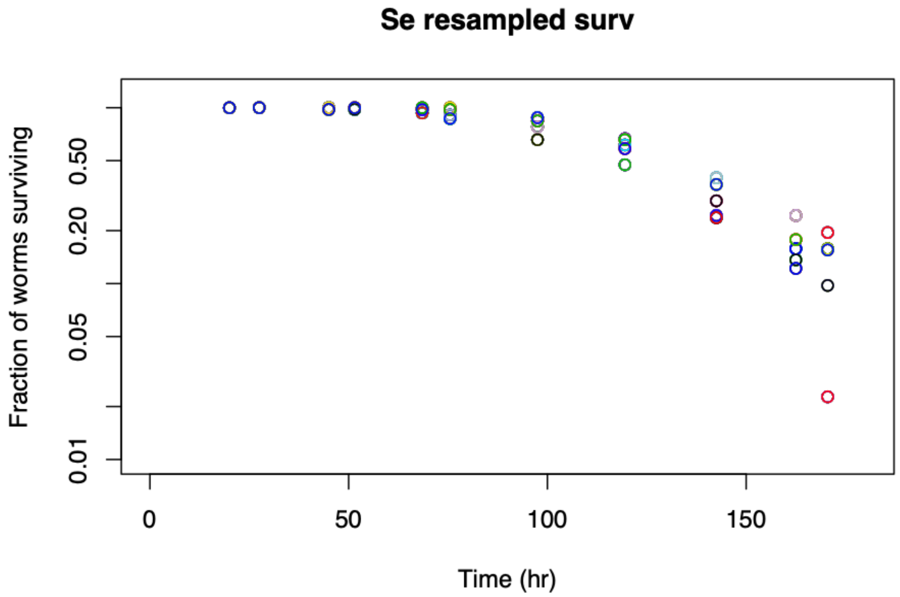

Bootstrap and fit for parameter instance for *Pa*.

```
pa_find_par_instance <- function () {
  # Return parameter instance.

  pa_growth_norm_inst <- resample_norm_growth_curve(pa_growth, KPa)
  pa_surv_inst <- resample_surv_curve(pa_dfs)

  pa_minimize_me2 <- function (pars) {
    # v2: works on resampled growth curve.
    # Objective function.
    # Take colonization rates and growth rate.
    # Return total error between model and data-sets paX_growth_norm
    # CAREFUL: in-coded data-sets.
    logr <- pars[[1]]
    logc <- pars[[2]]
    logd <- pars[[3]]

    err_growth <- get_growth_model_error(logr, logc, pa_growth_norm_inst)
    err_surv <- get_surv_model_error(logr, logc, logd, pa_surv_inst)
    total_err <- err_growth + err_surv
    return(as.double(total_err))
  }
  # print(pa_surv_inst)
  ans <- optim(par=pa_ans$par,
        fn=pa_minimize_me2,
        method = "Nelder-Mead")


  # print(exp(ans$par))
```

```r
  return(ans)
}
```

Bootstrap and fit for single parameter instance for *Sm*.

```r
sm_find_par_instance <- function () {
  # Return parameter instance.

  sm_growth_norm_inst <- resample_norm_growth_curve(sm_growth, KSm)
  sm_surv_inst <- resample_surv_curve(sm_dfs)

  sm_minimize_me2 <- function (pars) {
    # v2: works on resampled growth curve.
    # Objective function.
    # Take colonization rates and growth rate.
    # Return total error between model and data-sets paX_growth_norm
    # CAREFUL: in-coded data-sets.
    logr <- pars[[1]]
    logc <- pars[[2]]
    logd <- pars[[3]]

    err_growth <- get_growth_model_error(logr, logc, sm_growth_norm_inst)
    err_surv <- get_surv_model_error(logr, logc, logd, sm_surv_inst)
    total_err <- err_growth + err_surv
    return(as.double(total_err))
  }

  ans <- optim(par=sm_ans$par,
        fn=sm_minimize_me2,
        method = "Nelder-Mead")

  # print(exp(ans$par))
  return(ans)
}
```

Bootstrap and fit for single parameter instance for *Se*.

```r
se_find_par_instance <- function () {
  # Return parameter instance.

  se_growth_norm_inst <- resample_norm_growth_curve(se_growth, KSe)
  se_surv_inst <- resample_surv_curve(se_dfs)

  se_minimize_me2 <- function (pars) {
    # v2: works on resampled growth curve.
    # Objective function.
    # Take colonization rates and growth rate.
    # Return total error between model and data-sets paX_growth_norm
    # CAREFUL: in-coded data-sets.
    logr <- pars[[1]]
    logc <- pars[[2]]
    logd <- pars[[3]]

    err_growth <- get_growth_model_error(logr, logc, se_growth_norm_inst)
    err_surv <- get_surv_model_error(logr, logc, logd, se_surv_inst)
```

```r
    total_err <- err_growth + err_surv
    return(as.double(total_err))
  }

  ans <- optim(par=se_ans$par,
               fn=se_minimize_me2,
               method = "Nelder-Mead")

  # print(exp(ans$par))
  return(ans)
}
```

Bootstrap to obtain distributions of parameters for *Pa*

```r
pa_show_histograms <- function() {
  ## Generate distributions
  ds <- vector()
  cs <- vector()
  rs <- vector()
  for (i in 1:500) {
    ans <- pa_find_par_instance()
    rs <- c(rs, exp(ans$par[[1]]))
    cs <- c(cs, exp(ans$par[[2]]))
    ds <- c(ds, exp(ans$par[[3]]))
  }
  ## Compute s.e.m.
  r_sem <- round(sd(rs), digits = 4)
  c_sem <- round(sd(cs), digits = 5)
  d_sem <- round(sd(ds), digits = 5)
  ## Compute median
  r_val <- round(median(rs), digits = 4)
  c_val <- round(median(cs), digits = 5)
  d_val <- round(median(ds), digits = 5)
  ## Plot histograms
  hist(rs, main=paste('Pa growth rate. val:', r_val, "+/-", r_sem))
  hist(cs, main=paste('Pa col. rate. val:', c_val, "+/-", c_sem))
  hist(ds, main=paste('Pa lethality val:', d_val, "+/-", d_sem))
}
pa_show_histograms()
```

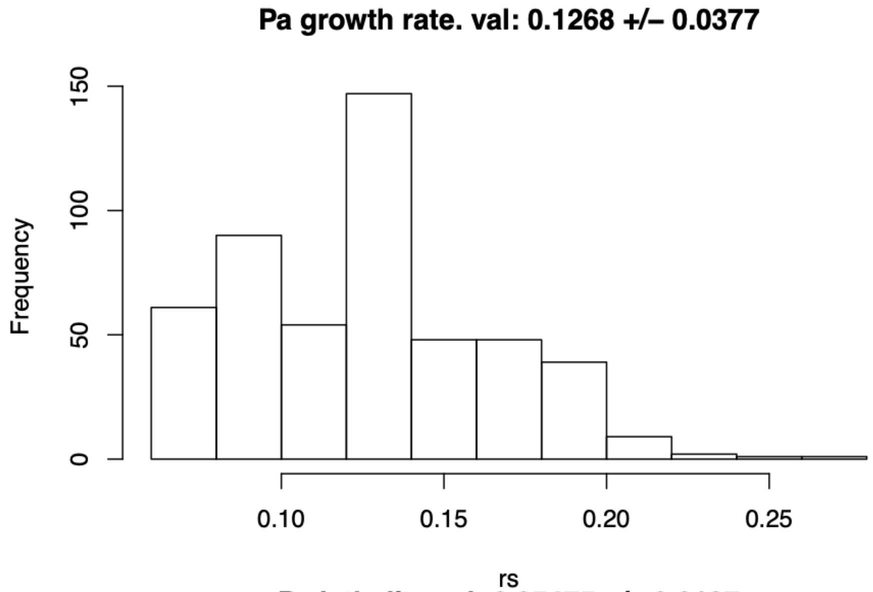

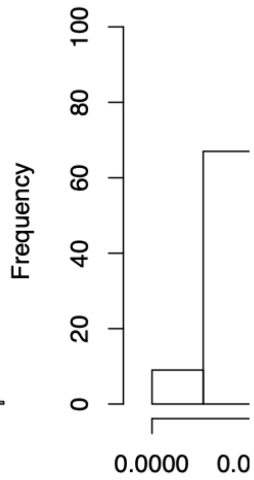

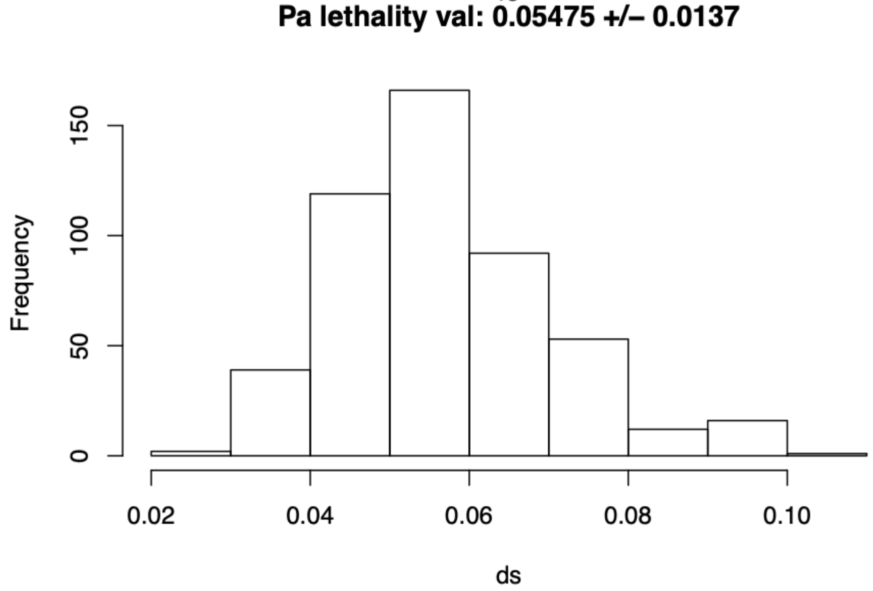

Bootstrap to obtain distributions of parameters for *Sm*

```
sm_show_histograms <- function() {
  ## Generate distributions
  ds <- vector()
  cs <- vector()
```

```
  rs <- vector()
  for (i in 1:500) {
    ans <- sm_find_par_instance()
    rs <- c(rs, exp(ans$par[[1]]))
    cs <- c(cs, exp(ans$par[[2]]))
    ds <- c(ds, exp(ans$par[[3]]))
  }
  ## Compute s.e.m.
  r_sem <- round(sd(rs), digits = 5)
  c_sem <- round(sd(cs), digits = 5)
  d_sem <- round(sd(ds), digits = 5)
  ## Compute median
  r_val <- round(median(rs), digits = 5)
  c_val <- round(median(cs), digits = 5)
  d_val <- round(median(ds), digits = 5)
  ## Plot histograms
  hist(rs, main=paste('Sm growth rate. val:', r_val, "+/-", r_sem))
  hist(cs, main=paste('Sm col. rate. val:', c_val, "+/-", c_sem))
  hist(ds, main=paste('Sm lethality val:', d_val, "+/-", d_sem))
}
sm_show_histograms()
```

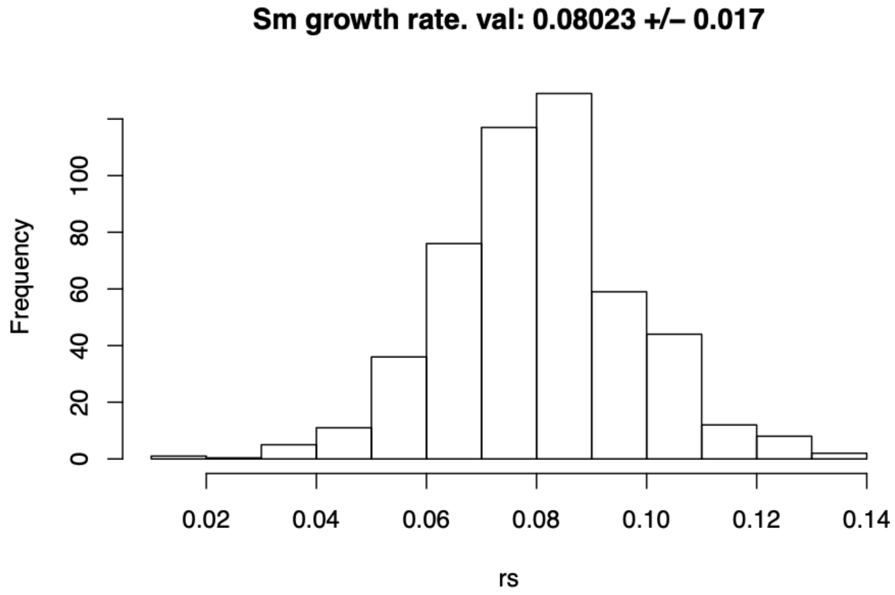

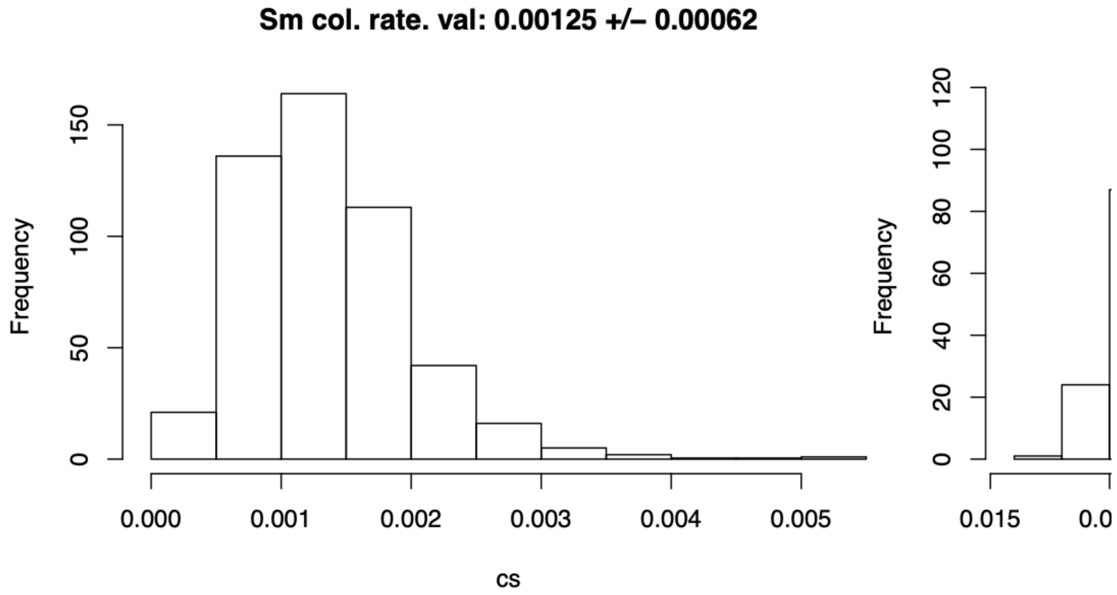

Bootstrap to obtain distributions of parameters for *Se*

```
se_show_histograms <- function() {
  ## Generate distributions
  ds <- vector()
  cs <- vector()
  rs <- vector()
  for (i in 1:500) {
    ans <- se_find_par_instance()
    rs <- c(rs, exp(ans$par[[1]]))
    cs <- c(cs, exp(ans$par[[2]]))
    ds <- c(ds, exp(ans$par[[3]]))
  }
  ## Compute s.e.m.
  r_sem <- round(sd(rs), digits = 5)
  c_sem <- round(sd(cs), digits = 5)
  d_sem <- round(sd(ds), digits = 5)
  ## Compute means
  r_val <- round(median(rs), digits = 5)
  c_val <- round(median(cs), digits = 5)
  d_val <- round(median(ds), digits = 5)
  ## Plot histograms
  hist(rs, main=paste('Se growth rate. val:', r_val, "+/-", r_sem))
  hist(cs, main=paste('Se col. rate. val:', c_val, "+/-", c_sem))
  hist(ds, main=paste('Se lethality val:', d_val, "+/-", d_sem))
}
se_show_histograms()
```

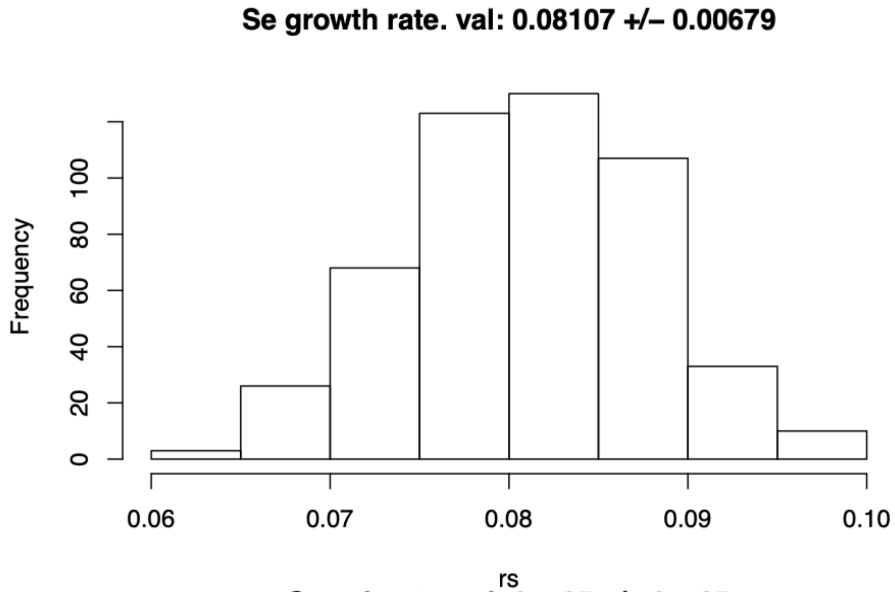

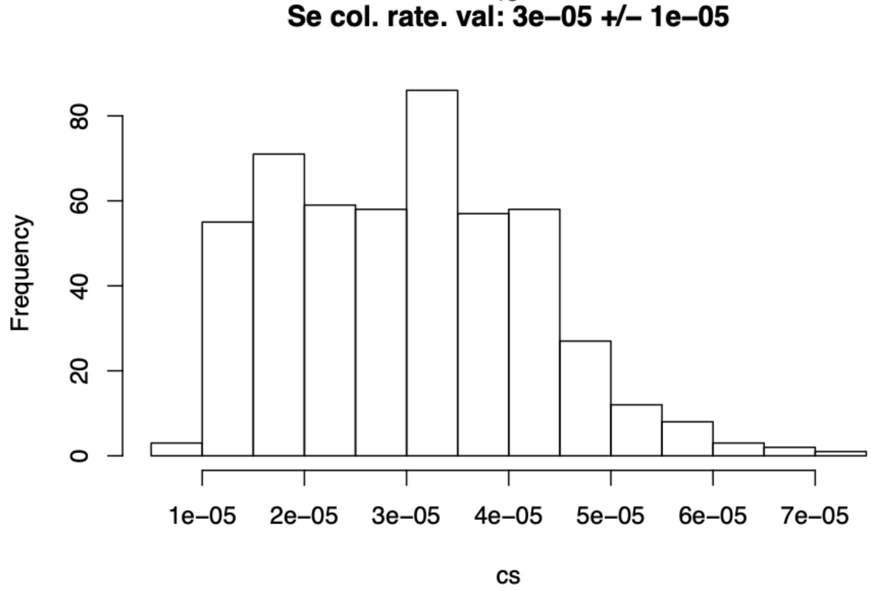

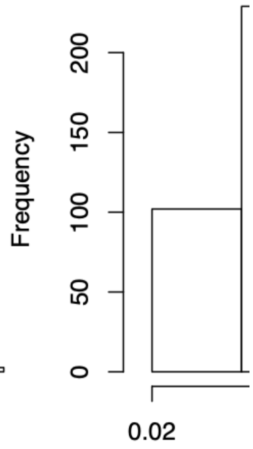

Rescale colonization rates by carrying capacities to be reported in paper. *Sm*:

```
c(0.00128 * KSm, 0.00058 * KSm)
```

```
## [1] 140.8  63.8
```

Pa:

```r
c(0.00042 * KPa, 0.00026 * KPa)
```

```
## [1] 117.6  72.8
```

Se:

```r
c(0.00005 * KSe, 0.00001 * KSe)
```

```
## [1] 85 17
```
