# Supplementary material for: Disentangling bacterial invasiveness from lethality in an experimental host‐pathogen system
Source: Mol Syst Biol. 2019 Jun 11;15(6):e8707. doi: 10.15252/msb.20188707 (PMC6558951; doi:10.15252/msb.20188707)
Supplement: Supplementary file 2 — Expanded View Figures PDF [file MSB-15-e8707-s002.pdf]

Expanded View Figures

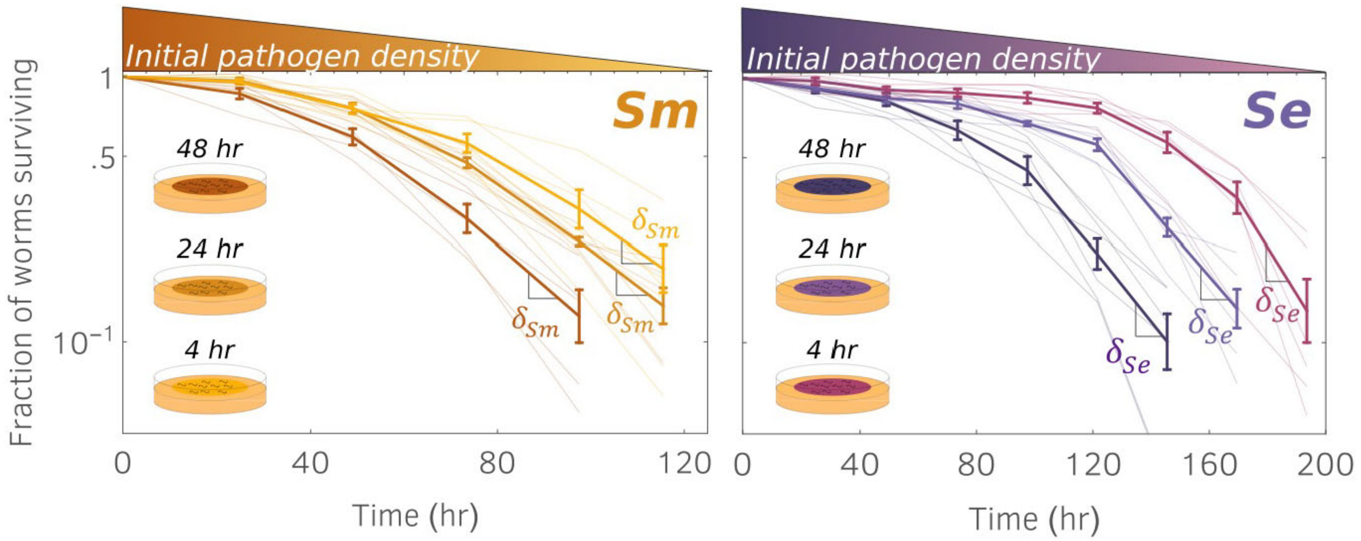

Figure EV1. Experiments confirm that for *Sm* and *Se*, the *LT50* is not robust against initial pathogen density, whereas the pathogen lethality δ is a robust indicator against the initial pathogen density fed to the hosts.
Survival kinetics for different initial pathogen densities, obtained by pre-incubating the pathogen lawns for different times prior to adding the nematodes. These panels show results similar to those in Fig 1A but for different pathogens. Here, solid lines connect experimental points which correspond to averages across replicas (thin lines). Error bars denote standard error. Lethalities are estimated and reported in Materials and Methods section from the main text (see also Supplementary Material: Statistical analysis) For both panels, we did not observe statistically significant differences between mean lethalities across different initial pathogen densities, as determined by one-way ANOVA (left panel: $F(2,14) = 0.47$, $P = 0.64$, right panel: $F(2,15) = 0.18$, $P = 0.84$).

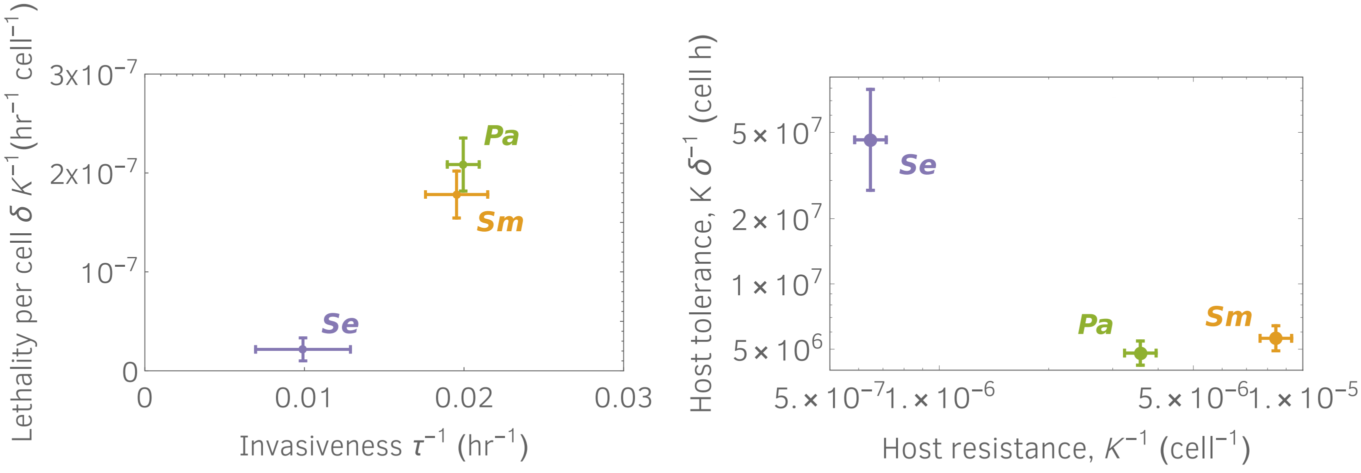

Figure EV2. Bacterial virulence for the three analyzed pathogens is revealed by means of several indicators.
Left panel shows invasiveness (the inverse of the time taken to enter the exponential phase) and pathogen lethality per cell, estimated from the data in Fig 1B (Dataset EV1). Lethality per cells is positively correlated to invasiveness. Overall, *Pa* and *Sm* are more virulent than *Se*. Error bars here indicate standard deviations. Right panel shows that hosts are more resistant, but less tolerant, to highly virulent pathogens. To define resistance and tolerance, we follow [7].

 © 2019 The Authors

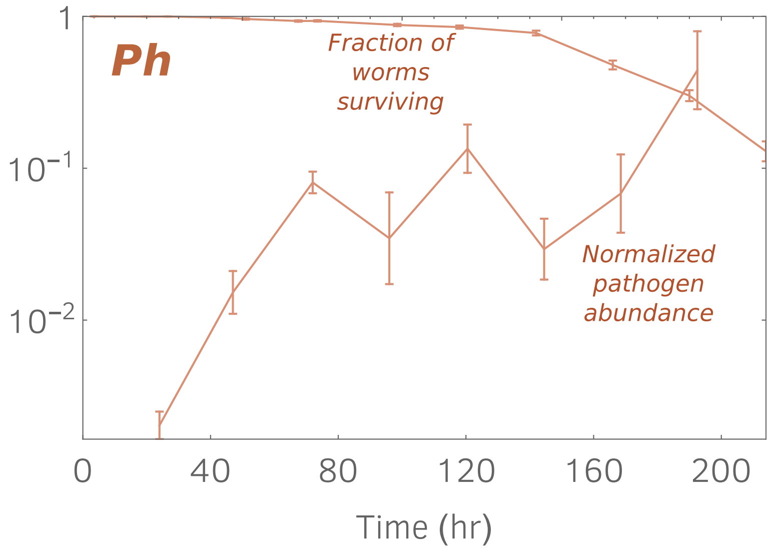

Figure EV3. Our theory does not apply to non-pathogenic bacterial species such as *Pseudomonas chlororaphis* (*Ph*).
We repeated our assays by exposing a population of worms to a lawn of the non-virulent bacterium *P. chlororaphis*. The figure shows data for the survival function (solid brown line) and the pathogen abundance curve normalized by $K_{Ph} = 3 \times 10^5$. Dissimilarly from the pathogens examined in the main text, we cannot identify an exponential phase in the survival function which corresponds to constant pathogen load. Solid lines connect experimental points.

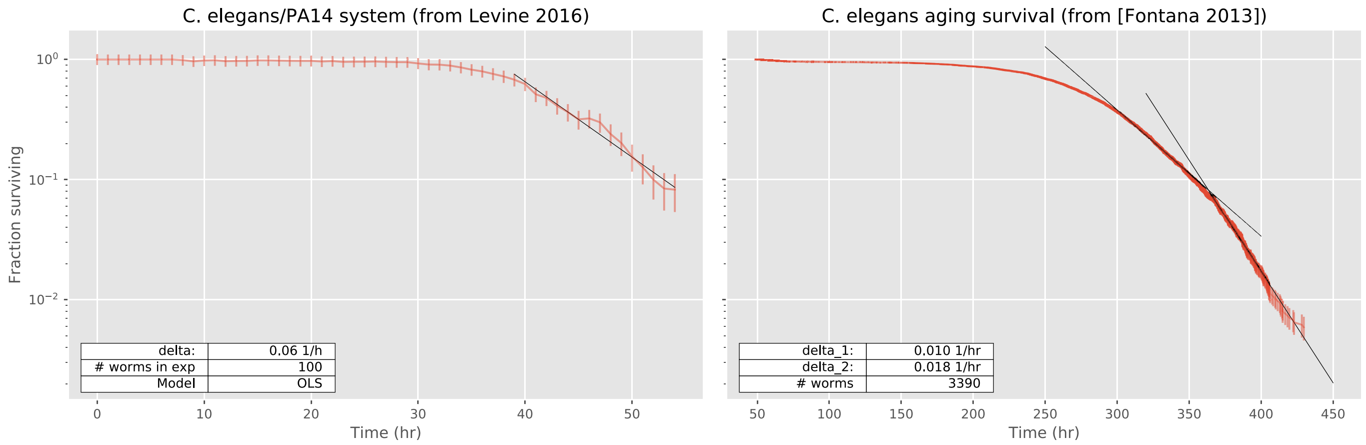

Figure EV4. Analysis of experimental data from two other studies suggests that exponential phase is observed in host–pathogen dynamics, whereas a more complex scenario arises in senescence.
Left: Survival kinetics for the *Caenorhabditis elegans*/*Pseudomonas aeruginosa* system, measured using *HandKAchip*—Hands-Free Killing Assay on a Chip (data from Erel Levine's Lab, 2016). The survival kinetics exhibits an exponential phase, consistently with our prediction, with exponent δ 0.06/h. Right: Survival kinetics of *C. elegans* wild-type population due to senescence from Stroustrup (2013). The survival kinetics, observed for a much larger time range than in our experiments, decay in a non-linear way, which is approximately exponential when restricted to certain time sub-intervals (e.g., two linear fits shown in figure). For age-induced death, the overall death rate of the host population is smaller than those observed in our pathogen-induced mortality experiments. A single replicate for each panel with Poissonian errors (for both panels)

© 2019 The Authors
