# Supplementary material for: Disentangling bacterial invasiveness from lethality in an experimental host‐pathogen system
Source: Mol Syst Biol. 2019 Jun 11;15(6):e8707. doi: 10.15252/msb.20188707 (PMC6558951; doi:10.15252/msb.20188707)
Supplement: Supplementary file 3 — Dataset EV1 [file MSB-15-e8707-s003.docx]

| **Dataset EV1** | ***Pa (48 hr)*** | ***Pa (24 hr)*** | ***Pa (4 hr)*** |
| --- | --- | --- | --- |
| Lethality time 50%, $50$ | $55hr$ | $80hr$ | $95hr$ |
| Pathogen lethality, $\delta$ | $0.062\pm0.007{hr}^{-1}$ | $0.053\pm0.003{hr}^{-1}$ | $0.052\pm0.006{hr}^{-1}$ |
| Pathogen growth rate, $r$ | $0.08\pm0.01{hr}^{-1}$ | $0.08\pm0.01{hr}^{-1}$ | $0.08\pm0.01{hr}^{-1}$ |
| Pathogen colonization rate, $c$ | $1031\pm120{hr}^{-1}$cells | $236\pm48{hr}^{-1}$cells | $94\pm14{hr}^{-1}$cells |
| Carrying capacity, $K$ | $2.8\times{10}^{5}$ cells | $2.8\times{10}^{5}$ cells | $2.8\times{10}^{5}$ cells |
| Colonization threshold, $N$ | $13000\pm2000$ cells | $3000\pm700$ cells | $1175\pm230$ cells |
| Colonization time, $\tau_{c}$ | $9\pm1hr$ | $9\pm1hr$ | $9\pm1hr$ |
| Replication time, $\tau_{r}$ | $28\pm2hr$ | $48\pm5hr$ | $59\pm6hr$ |
| Invasiveness, $\tau$ | $37\pm4hr$ | $56\pm6h$r | $68\pm7hr$ |

a

**Dataset EV1** refers to *Pseudomonas aeruginosa* experiments displayed in Figure 1A and Figure 4C.
