# Supplementary material for: Disentangling bacterial invasiveness from lethality in an experimental host‐pathogen system
Source: Mol Syst Biol. 2019 Jun 11;15(6):e8707. doi: 10.15252/msb.20188707 (PMC6558951; doi:10.15252/msb.20188707)
Supplement: Supplementary file 4 — Dataset EV2 [file MSB-15-e8707-s004.docx]

| **Dataset EV2** | ***Pa*** | ***Sm*** | ***Se*** |
| --- | --- | --- | --- |
| Lethality time 50%, $50$ | $60hr$ | $70hr$ | $120hr$ |
| Pathogen lethality, $\delta$ | $0.057\pm0.014{hr}^{-1}$ | $0.024\pm0.006{hr}^{-1}$ | $0.028\pm0.007{hr}^{-1}$ |
| Pathogen growth rate, $r$ | $0.13\pm0.037{hr}^{-1}$ | $0.082\pm0.017{hr}^{-1}$ | $0.079\pm0.006{hr}^{-1}$ |
| Pathogen colonization rate, $c$ | $118\pm73{hr}^{-1}$cells | $132\pm64{hr}^{-1}$cells | $56\pm17{hr}^{-1}$cells |
| Carrying capacity, $K$ | $2.8\times{10}^{5}$ cells | $1.1\times{10}^{5}$ cells | $1.7\times{10}^{6}$ cells |
| Colonization threshold, $N$ | $910\pm620$ cells | $1610\pm850$ cells | $710\pm220$ cells |
| Colonization time, $\tau_{c}$ | $5\pm2hr$ | $9\pm2hr$ | $9\pm1hr$ |
| Replication time, $\tau_{r}$ | $39\pm10hr$ | $42\pm9hr$ | $90\pm7hr$ |
| Invasiveness, $\tau$ | $44\pm11hr$ | $51\pm10h$r | $98\pm8hr$ |

**Dataset EV2** refers to multi-pathogen experiments displayed in Figure 1B/C, Figure 3B/C and Figure 4B.
